# Affordable, cleanroom-free millifluidic production of targeted lipid nanocarriers *via* additive manufacturing

**DOI:** 10.1039/d3lc00995e

**Published:** 2026-01-07

**Authors:** Callum D. Hay, Suchaya M. Mahutanattan, Colin P. Pilkington, Miguel Paez-Perez, Kimberly A. Kelly, Yuval Elani, Marina K. Kuimova, Nicholas J. Brooks, Michela Noseda, James W. Hindley, Oscar Ces

**Affiliations:** a Department of Chemistry, Molecular Science Research Hub, Imperial College London 82 Wood Lane London W12 0BZ UK j.hindley14@imperial.ac.uk o.ces@imperial.ac.uk; b Institute of Chemical Biology, Imperial College London Molecular Sciences Research Hub London W12 0BZ UK; c Imperial Centre for Translation and Experimental Medicine, Imperial College London Hammersmith Campus London SW7 2AZ UK; d Department of Biomedical Engineering, University of Virginia Charlottesville VA 22908 USA; e Department of Chemical Engineering, Imperial College London Exhibition Road London SW7 2BX UK; f fabriCELL, Imperial College London and King's College London London W12 0BZ UK

## Abstract

Lipid nanocarriers utilise the self-assembly of amphiphilic molecules to generate particle formulations capable of drug encapsulation and dynamic interactions with user-defined cell types, enabling applications within targeted therapeutic delivery. This offers increased bioavailability, stability, and reduced off-target effects, with the promise of application to numerous cell types and consequently, diseases. Here, we have developed a highly accessible, cleanroom-free method for the fabrication of poly(methyl methacrylate) millifluidic vertical flow focusing (VFF) devices *via* laser cutting, multilayered solvent and heat-assisted bonding. We demonstrate that these can be used for one-step production of targeted lipid nanocarriers *via* the production of cardiomyocyte-targeting vesicle nanoparticles loaded with the hydrophobic drug menadione. We characterise vesicle size using dynamic light scattering (DLS) and cryogenic transmission electron microscopy (cryo-TEM), whilst also probing the membrane viscosity of vesicles produced *via* flow-focusing for the first time using molecular rotors. Finally, we apply cardiomyocyte-targeting, menadione-loaded vesicles to H9C2 tissue culture demonstrating significant inhibition of cell viability *via* targeted delivery, showcasing the potential of our device to produce formulations for therapeutic delivery. As a flow-based method, VFF can facilitate rapid formulation investigation and produce large sample volumes for cell-based validation studies, whilst avoiding inter-batch sample variation. Furthermore, the accessible nature of this VFF approach will help to democratise millifluidics, facilitating the wider adoption of flow-based production methods to develop nanomedical formulations.

## Abstract 2

Affordable, clean room-free PMMA vertical flow-focusing device assembly enables the production of targeted lipid nanocarriers, democratising millifluidic nanocarrier production.

## Graphical abstract

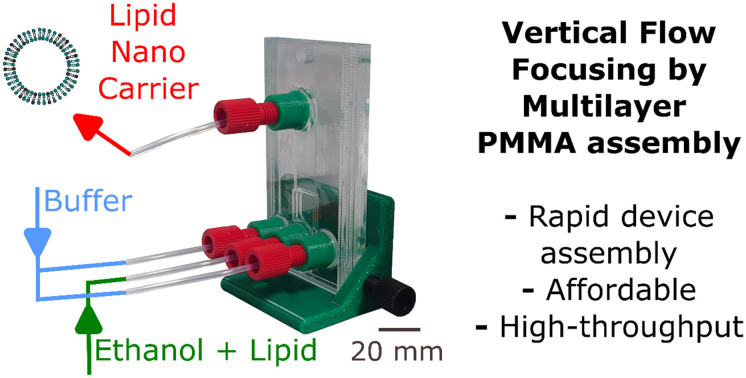

## 1. Introduction

Nanomedical delivery systems have shown great promise in improving the pharmacokinetics of drug delivery compared to conventional formulations.^1^ Such nanocarriers can be produced from molecular building blocks such as phospholipids,^2^ offering environments for the loading of drugs, and scaffolds that can be functionalised to generate vectors with stimuli-responsive^3^ and/or targeting capabilities.^4^ Phospholipids can be used as building blocks for constructing artificial membranes which aim to replicate aspects of biological membranes,^1^ and are amphiphilic molecules comprised of a hydrophilic head group and hydrophobic tail region. This structure facilitates the self-assembly of phospholipids into vesicles that resemble a spherical cellular membrane.^2^ When applied as bioinspired nanocarriers, phospholipids offer self-assembly, biomimicry, and functionalization through the incorporation of synthetic lipids, proteins, and other membrane-associated molecules such as deoxyribonucleic acid nanostructures.^3–5^

**Fig. 1:**
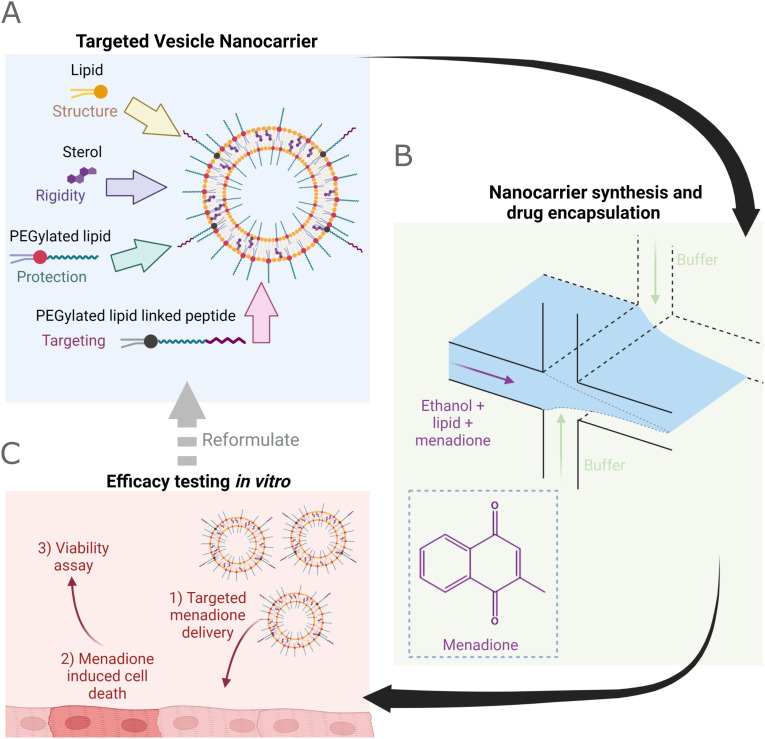
Rapid nanocarrier formulation development using an accessible and affordable VFF device. A) Cartoon representation of lipid vesicle nanocarriers functionalized for therapeutic delivery *in vivo* showing lipid, sterol, PEGylated lipid, and targeting moieties. B) Proposed synthesis of nanocarriers containing the hydrophobic drug molecule menadione, pictured bottom left, using the vertical flow focusing process. C) Efficacy testing of cardiac targeting nanocarriers *in vitro* through a viability assay.

Within the field of therapeutic delivery small unilamellar vesicles (20–100 nm) dominate, where miniaturization is utilized in conjunction with poly(ethylene glycol) (PEG) functionalized lipids (PEGylated lipid) to reduce the rapid uptake by the reticuloendothelial system resulting in improved circulation times *in vivo* (Fig. 1A).^6–9^ In addition to enhanced circulation, compared to free drug molecules, therapeutic vesicles offer scope for ‘active’ targeting methods that go beyond ‘passive’ targeting to improve accumulation within localized injuries or tissues.^9–16^ Typically, vesicles aim to encapsulate small molecule drugs (<340 Da (ref. 17)) that are either hydrophilic (within the aqueous core) or hydrophobic (within the lipid leaflet), facilitating delivery of labile and lipophilic drug molecules.^10,13,18–20^

An example hydrophobic molecule is menadione (vitamin K_3_) which is a metabolite of vitamin K (Fig. 1B). Menadione acts as a source of oxygen radical species and within cardiomyocytes depletes cellular glutathione levels, increases intracellular Ca^2+^, and incites lipid peroxidation.^21–27^ The generation of reactive oxygen species (ROS) at low concentrations (∼2 μM menadione^28^) produces redox-active signaling messengers and at high concentrations, induces cellular death.^23,25–27^ Beyond serving as a model for oxidative damage and subsequent apoptosis, menadione has exhibited potential as a chemotherapeutic agent.^28–33^ However, whilst the hydrophobicity of menadione has limited its application as a potential drug to date, incorporation within the lipid bilayer of cardiac-targeting vesicle nanocarriers may yet provide an effective means for menadione therapeutic application whilst reducing off-target oxidative stress and improving patient outcomes (Fig. 1C).^30,31,33^ To realize such applications scalable means for lipid nanocarrier synthesis are essential.

Within an academic setting, the production of small unilamellar vesicles has been dominated by the extrusion process. Extrusion is a batch-based method that operates through the cycling of a hydrated lipid film through a membrane of defined pore size.^34–37^ Size control within extrusion is achieved by the application of manufactured membranes and determined by the discrete pore sizes available. More recently, the method of microfluidic hydrodynamic flow focusing (MHF) was reported for the controlled production of small to large unilamellar vesicles (100–300 nm,^38^ Fig. S1A).^38–44^ Microfluidic hydrodynamic flow focusing involves the co-flow of a lipid in ethanol solution with an aqueous buffer. When co-flowed, ethanol is diluted, and lipid controllably self-assembled into lipid vesicles, by a purported disc-like micelle intermediate (Fig. S1C).^39,44^ By varying the flow rate ratio (FRR), the ratio of the total aqueous flow rate to the ethanol flow rate, size control can be achieved.^38,39^ The total flow rate (TFR), is the sum of ethanol and aqueous flow rates, a value that affects both the residence time within devices and the production rate of particles.^38^

Limited examples of MHF application to drug delivery exist, which in part is the result of the micron scale channel sizes (≤200 μm) and relatively low flow rates (μL min^−1^) resulting in low particle throughput.^45–47^ Vertical flow focusing (VFF) is an adaption of MHF where the aspect ratio (AR) of the channel is dramatically increased (From AR ≈ 1 to AR > 10, Fig. S1B).^47–49^ VFF exploits high AR to retain nanoliposome production whilst increasing throughput (mL min^−1^) from the application of a larger channel cross-sectional area. Inherently, VFF devices are millifluidic and the design is highly amenable to cleanroom-free fabrication, resulting from the large channel dimensions. Currently reported fabrication methods for VFF include deep reactive ion etching and hot embossing,^41,47,48^ which can require specialist equipment for chip fabrication, reducing the accessibility of VFF device production. The increased particle synthesis rate of VFF makes such devices suitable for nanocarrier production at the preclinical scale, from simple liposomes to synthetic exosome mimics.^52^

Within this work, we adopt a solvent-mediated bonding approach to produce the first example of a millifluidic VFF device from a multilayer poly(methyl methacrylate) (PMMA) design, which is affordable, accessible, and enables the addition of further functionality such as dialysis-on-chip.^47^ We then use the VFF device for flow-based production (at rates up to 2.6 mL min^−1^) of a range of particles, from single-component lipid vesicles to cardiac-targeting, menadione-loaded formulations, characterizing their properties *via* light-scattering, cryo-EM, and spectroscopic microviscosity data (Fig. 1B). We note that the characterization of membrane microviscosity using molecular rotors is the first application of this method to hydrodynamic flow focusing, facilitating analysis of the effect of residual ethanol (or other organic solvent) incorporation within the bilayer, which can affect key membrane properties including permeability. Finally, the application of vesicle nanoparticles geared for the drug delivery of menadione to C2C12 cardiomyocytes is assessed by a viability assay, demonstrating the feasibility of peptide-mediated delivery of menadione (Fig. 1C). This indicates the potential of multilayer PMMA VFF devices to produce formulations suitable for biomedical application and marks the first formulation of menadione within a peptide-functionalized lipid vesicle, facilitating re-evaluation of the drug as a chemotherapeutic.^29^

## 2. Results and discussion

### 2.1. Vertical flow-focusing device fabrication and characterisation

**Fig. 2:**
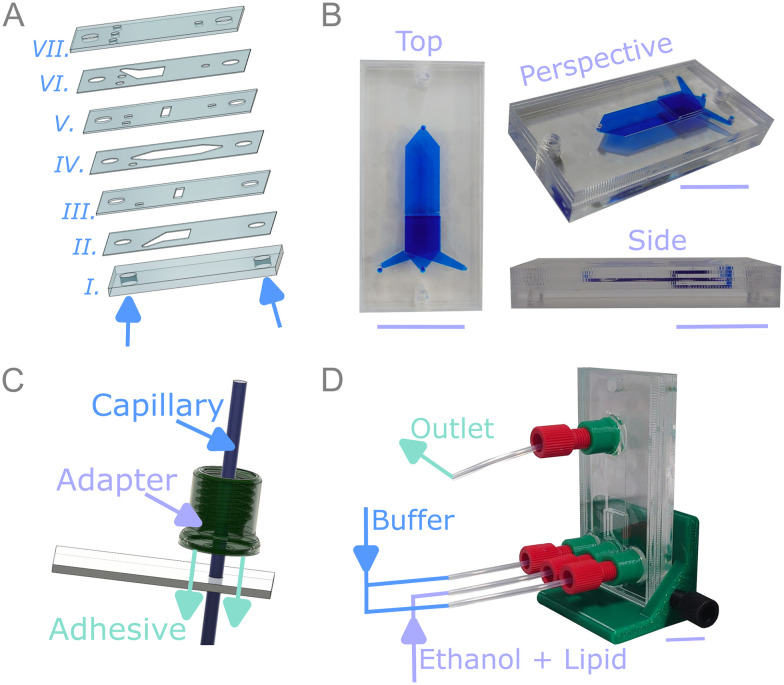
Illustrations and images of the vertical flow-focusing device and manufacture. A) Exploded view of the seven-layer PMMA structure depicting the 5 mm thick base (part I) and 1 mm thick channel structures (parts II–VI), channel connectors (parts III and V), and top (part VII) (not to scale). B) PMMA structure post-bonding highlighting fluidic sealing (scale bars ≈ 20 mm). C) Process of fitting adapter alignment and adhesion to the sealed PMMA device where the adapter is aligned using a rigid capillary and adhered using quick set epoxy resin (not to scale). D) Complete device with fitting adapters (green), shown upright in a 3D printed mount (green) and fitted with PEEK fittings (red), directions of experimental fluid flow are highlighted on the device (scale bar ≈ 20 mm).

The vertical flow-focusing device was fabricated from a seven-layer PMMA assembly depicted in Fig. 2A, S2 and S3. The device featured a 5 mm thick base (part I) where the holes used to align the PMMA stack are highlighted in Fig. 2A. The channel structures (parts II–VI) were comprised of 1 mm PMMA, connected using rectangular channels (parts III and V), and sealed by an inlet segment (part VII) that were all cut in 1 mm PMMA. Briefly, the device was bonded using an aluminum heating block and applying a low-warping, ethanol-assisted heat bonding method, bonding at 70 °C for 120 minutes.^50^ The bonding process resulted in the millifluidic device shown in Fig. 2B, where the flow-focusing junction is shown in the side view and the exit channel (10 mm × 1 mm × 25 mm, width × depth × length) and post-flow focusing is shown in the top view.

The PMMA device was made compatible with poly(ether ether ketone) (PEEK) microfluidic fittings using a 3D printed glycol-modified poly(ethylene terephthalate) (PET-G) fitting adapter, which was aligned using a rigid capillary and subsequently adhered to the PMMA device depicted by the process in Fig. 2C. The final device depicted in Fig. 2D was secured in an upright orientation using a 3D printed mount such that the direction of flow focusing opposed gravity. Millifluidic device orientation is reported to be crucial to prevent stream deflection within the device, we find the deviation of the central ethanol stream in this device, when oriented perpendicular to gravity, results in higher polydispersity of liposome distributions.^49^ Overall, the device features an accessible, rapid cleanroom-free fabrication with an estimated material cost per device of £1.30 (eqn (S1)). Without laser cutting and 3D printing access, cutting and printing services could be employed such that the fabrication purely requires hotplate accessibility. Application of the staged fabrication is amenable to additional functionality such as dialysis on-chip and continuous lipid formulation adaptation through mixing on-chip.^47^ Finally, the use of high optical clarity PMMA could facilitate simultaneous analysis by SAXS or fluorescent microscopy.^51^

**Fig. 3:**
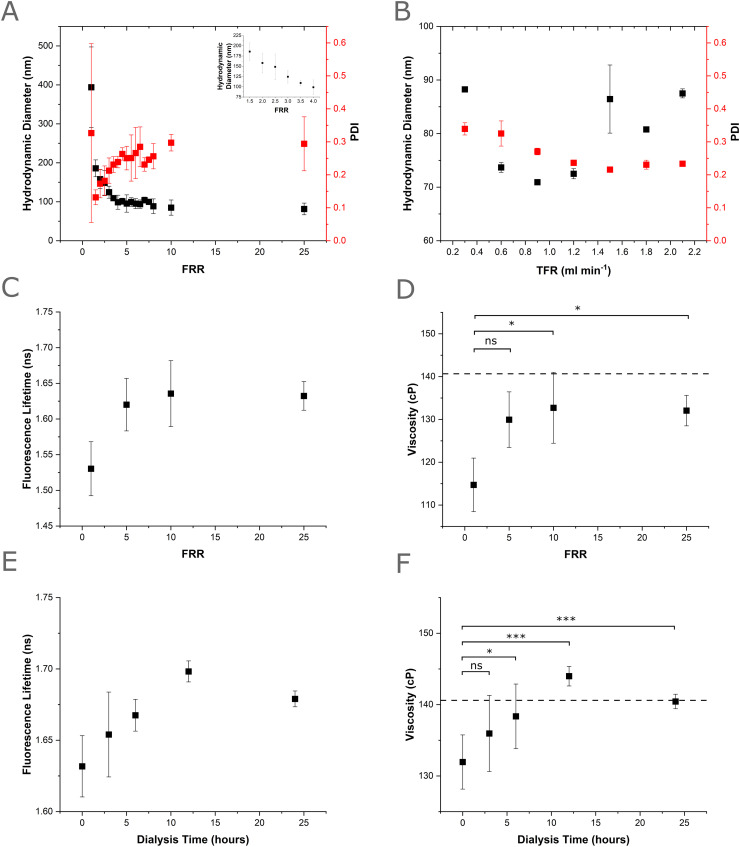
Production of DOPC nanocarriers *via* the PMMA VFF device (5.0 mg ml^−1^ DOPC lipid in ethanol and a PBS buffer phase). A) Effect of increasing FRR on hydrodynamic diameter and PDI of produced DOPC nanocarriers (*n* = 3, error = ±1 sd). The inset shows a magnified view of the hydrodynamic diameter for FRR values between 1.5 and 4. Size distributions for all FRR are shown in Fig. S3. B) Effect of increasing TFR on hydrodynamic diameter PDI of produced DOPC nanocarriers (*n* = 3, error = ±1 sd). Size distributions for all TFR are shown in Fig. S4. C) Fluorescence lifetime measurements of BC10 rotor (0.5 mol%) embedded in DOPC nanocarriers produced with varying FRR (*n* = 3, error = ±1 sd). D) Membrane microviscosity values for DOPC nanocarriers produced with varying FRR derived from lifetime measurements in Fig. 4C (*n* = 3, error = ±1 sd). Dashed line represents the microviscosity of an ethanol-free sonicated DOPC vesicle control. *P* values calculated using one-way ANOVA, Tukey *post hoc* test. ns = *p* > 0.05, * = 0.05 > *p* > 0.01. E) Fluorescence lifetime of BC10 rotor embedded in DOPC nanocarriers produced at FRR 5, TFR = 0.6 mL min^−1^ and exposed to increasing dialysis periods (*n* = 6, error = ±1 sd). F) Membrane microviscosity values for DOPC nanocarriers produced at FRR 5, TFR = 0.6 mL min^−1^ and exposed to increasing dialysis periods, derived from lifetime measurements in Fig. 4E (*n* = 6, error = ±1 sd). Dashed line represents the microviscosity of an ethanol-free sonicated DOPC vesicle control. *P* values calculated using one-way ANOVA, Tukey *post hoc* test. ns = *p* > 0.05, * = 0.05 > *p* > 0.01, ** = 0.1 > *p* > 0.01, *** = *p* < 0.01.

The efficacy of the VFF device at producing nano-sized liposomes was evaluated using the lipid 1,2-dioleoyl-*sn-glycero*-3-phosphocholine (DOPC) in the ethanol phase (5.0 mg mL^−1^, 6.35 mM). Increasing the FRR (1–25, Table S1) was found to affect the resultant mean vesicle size from 393.9 ± 103.4 nm (FRR 1, TFR = 0.2 mL min^−1^) to 81.53 nm ± 14.96 nm (FRR 25, TFR = 2.6 mL min^−1^). Within this FRR range, the greatest change in size was observed between FRRs 1.5–4 (185.8 ± 21.6 nm–98.75 ± 18.0 nm), with a gradual reduction in size observed between FRR 4–25 (summary data shown in Fig. 3A and size distributions shown in Fig. S4). After initially recording high polydispersity index (PDI) at FRR 1 (0.33 ± 0.27), we saw a large reduction in the PDI of produced liposomes from FRR 1.5 (0.13 ± 0.02), which then slowly increased to a plateau around FRR4 (0.24 ± 0.02), with the largest recorded PDI for FRR 25 (0.30 ± 0.03). Similar trends of decreasing particle diameter and increasing PDI with increasing FRR have been observed in previously designed, vertical flow focusing devices^52^ demonstrating a good level of control over nanocarrier size with respect to FRR.

Next, the effects of increasing TFR at FRR 5 was evaluated (summary data shown in Fig. 3B and size distributions shown in Fig. S5). Here the hydrodynamic radius was shown to decrease with increasing TFR, with vesicle diameters of 88.25 nm ± 0.47 nm observed at TFR = 0.3 mg ml^−1^ reducing to 72.5 nm ± 0.95 nm observed at TFR = 1.2 ml min^−1^. Above this TFR, the size increased back to its original value, with vesicle diameters of 87.50 nm ± 0.84 nm observed at TFR = 2.1 ml min^−1^. The PDI decreased from 0.34 ± 0.02 (TFR = 0.3 mL min^−1^) to 0.23 ± 0.003 (TFR = 2.1 mL min^−1^). Previous VFF devices have shown a reduction in vesicle size with increasing TFR but have not shown this type of parabolic behaviour. Interestingly, parabolic variation in PDI has been observed by Chen *et al.*, but in our case, we saw a progressive reduction in PDI with increasing TFR. Such trends are advantageous, as nanocarrier production rates (mL min^−1^) increase with increasing TFR, and for high-through throughput a combination of high TFR and low PDI is ideal. This initial DOPC sizing study demonstrates the applicability of the PMMA VFF device for the formation of vesicle nanocarriers with good size and PDI control within the explored FRR (1–25) and TFR ranges (0.3–2.1 mL min^−1^).

After confirming the VFF device could be used to produce vesicle nanocarriers, we investigated the potential effect of residual ethanol incorporation within the nanocarriers. Retaining residual solvent within the vesicle bilayer is a potential limitation of flow focusing microfluidics, and ethanol can readily incorporate into lipid bilayers, resulting in lateral expansion and bilayer thinning (which can impact drug loading and retention).^53^ To evaluate the ethanol impact on the membrane organization of nanocarriers formed with the VFF method, viscosity-sensitive membrane dyes, termed molecular rotors (MRs) were used. Compared to other approaches, the use of molecular rotors enables the high-throughput quantitative evaluation and imaging of microviscosity and/or molecular crowding of their surrounding environment.^54^ Upon excitation, relaxation of these dyes to the ground state proceeds *via* competing radiative and non-radiative decay pathways. The former is favoured when intramolecular motion of the MR is restricted – such as in a highly viscous environment – and, therefore, fluorescence intensity and lifetime is increased according to the Förster and Hoffmann equation:

1 log_10_ *F* = *c*_1_ + *c*_2_ log_10_ *η*

where *F* represents the fluorescence descriptor (*e.g.* lifetime), *η* the viscosity, and *c*_*i*_ are constants which can be derived by calibrating the MR's response in solutions of known viscosity.^55^ This relationship has been used to investigate the molecular organisation of both artificial and cellular lipid membranes, and to study the effect of physical and chemical stress on the bilayer's structure.^56–58^

Here, we use a boron dipyrromethene difluoride (BODIPY) based molecular rotor, BC10, to measure changes in the microviscosity of liposomes formed *via* VFF. Importantly, the readout from the BC10 probe has been shown to exhibit a direct correlation to the membrane's molecular architecture^59^ to measure changes in the membrane microviscosity caused by the presence of residual ethanol remaining from the VFF process.^56,60^ BC10 is added to the lipids in the ethanol solution at 0.5% mol, to avoid aggregation of the dye. The BC10 rotor locates within the tail region of the lipid bilayer upon vesicle assembly, facilitating reporting of membrane microviscosity by fluorescence lifetime of the rotor.^56^ The time-resolved decay traces of BC10 (Fig. S6) indicated a small increase in lifetime (Fig. 3C) and hence membrane microviscosity from 115 ± 6 cP to 133 ± 3 cP with increasing FRR (1 to 25) and TFR (0.2–2.6 mL min^−1^), with this increase observed from FRR 5, upon which the viscosity plateaued (Fig. 3D). This viscosity is slightly less than a sonicated vesicle control (140.6 ± 12.2 cP) indicating at FRR ≥ 5, low and consistent levels of ethanol are retained in the vesicle membranes. This result is consistent with previous findings which suggest alcohols lower the microviscosity of lipid membranes.^61^

We further evaluated the microviscosity of VFF-produced vesicles prepared at FRR 5 that were dialysed for 24 hours, aiming to reduce the levels of residual ethanol. We saw a progressive increase in fluorescence lifetime (Fig. 3E) and hence membrane viscosity (Fig. 3F) over the initial 12 hours, with measured viscosity of vesicles dialysed for 12 hours (144 ± 0.54 cP) and 24 hours (140.44 ± 0.22 cP) significantly higher (*p* < 0.01 based on ANOVA analysis) than undialysed vesicles produced on chip (132 ± 4.88 cP), and within the viscosity range of ethanol-free sonicated vesicles produced off chip (140.6 ± 12.2 cP). This indicates that 12 hours of dialysis is sufficient to reduce residual ethanol concentrations present in the vesicles to that of solvent-free membranes. To the best of our knowledge, this is the first time viscosity-sensitive dyes have been applied to evaluate the membrane integrity of liposomes produced by hydrodynamic flow focusing, offering a quantitative evaluation of the impact of ethanol. Application of this method could enable the rapid evaluation of membrane viscosity (and hence the extent of residual solvent) for a range of solvents applied in hydrodynamic flow focusing.^38–40^ Overall, the determination of the lipid vesicle microviscosity using BC10 suggests that for vesicle nanocarriers produced at FRR ≥ 5, the viscosity of vesicles produced on chip is slightly less than solvent free vesicles, and 12 hours of dialysis is required to achieve membrane microviscosity comparable to solvent-free samples.

### 2.2. Synthesis and characterization of cardiomyocyte-targeting nanocarriers

**Fig. 4:**
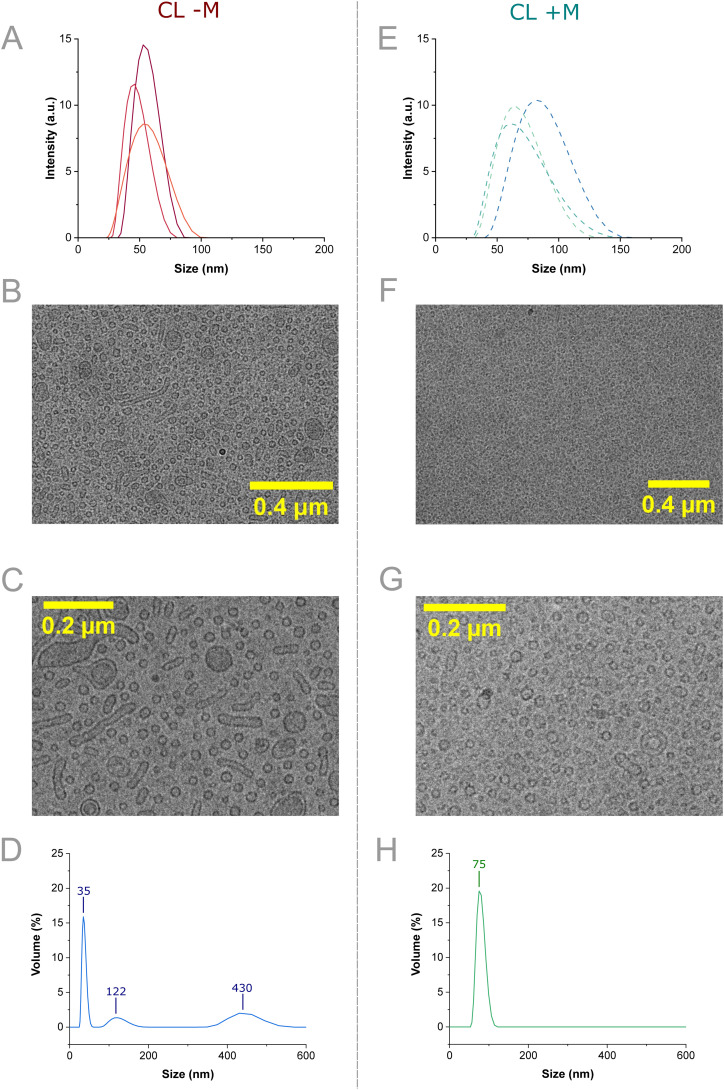
Characterization studies of VFF synthesized vesicle nanocarriers without menadione encapsulation (CL − M) and with menadione encapsulation (CL + M). A) Size against intensity plots achieved from CL formulation replicates, achieved by DLS (*n* = 3). B) Representative cryo-TEM image of the CL − M formulation showing populations of small vesicles, and worm-like vesicles. C) Higher magnification cryo-TEM images of a CL − M formulation showing populations of small unilamellar vesicles, multi-lamellar vesicles, and worm-like vesicles. D) MADLS size by volume plots for the CL − M cryo-TEM sample. E) Size against intensity plots achieved from CL + M formulation replicates encapsulating 70 μM menadione, achieved by DLS (*n* = 3). F) Representative cryo-TEM image of the CL + M sample showing a predominant population of small vesicles. G) Higher magnification cryo-TEM image of a CL + M sample showing small liposomes that were largely unilamellar. H) MADLS size by volume plots for the CL + M cryo-TEM sample.

Following the initial device application and vesicle characterization, the VFF device was utilized to produce a multicomponent formulation suitable for cellular delivery. The production of PEGylated vesicle compositions suited for drug delivery (47 : 46 : 7, DOPC : cholesterol : DSPE-PEG_2000_ mol%) using the VFF device was first characterized by DLS shown in Fig. 4A and S7. Sizing of the control formulation was measured for vesicles produced from the control formulation without menadione (CL − M, 47 : 46 : 7, DOPC : cholesterol : DSPE-PEG_2000_ mol%) and encapsulating menadione (70 μM) *via* incorporation within the ethanol stream (CL + M, 47 : 46 : 7 : 19, DOPC : cholesterol : DSPE-PEG_2000_ : menadione mol%), both at identical flow conditions (FRR = 10, TFR = 1.1 mL min^−1^). Menadione concentration was chosen from the results of a menadione titration, where 4-hour incubation of 70 μM menadione resulted in a ∼60% loss in H9C2 cell viability (Fig. S8). Both the average mean diameter, PDI and particle concentration of nanocarriers were shown to increase (52.8 ± 2.5 nm to 73.8 ± 5.1 nm, 0.091 ± 0.02 to 0.130 ± 0.01 and 3.12 ± 0.92 particles per ml to 5.05 ± 2.34 particles per ml respectively) with the inclusion of menadione (Fig. 4E and S7 and Table S3). The swelling supports that menadione encapsulation occurred within the lipid bilayer of the vesicles, which was expected from the hydrophobic nature of menadione and its inclusion at high molar percent in the lipid film. Despite vesicle swelling, samples remained below the 200 nm threshold size for drug delivery, demonstrating the applicability of our VFF device for producing nanoscale formulations suited for therapeutic validation.^62,63^

Following DLS sizing, CL − M was imaged using cryo-TEM, to establish the size, morphology, and lamellarity of the produced vesicles (Fig. 4B/C). The CL − M formulation exhibited a mixture of predominantly small unilamellar vesicles (<100 nm diameter) coexisting with wormlike vesicles (200–400 nm length) and larger unilamellar and multilamellar vesicles (≈200 nm diameter) (Fig. S9A and S7). Classifying the particles in CL − M samples, it can be observed that the majority of particles are either small vesicles (72%) or wormlike vesicles (25%), with a minority being larger vesicles (3%) (Fig. S9C). Further sizing analysis shows average particle dimensions of 76.1 ± 46.3 nm length × 16.3 ± 2.4 nm width for wormlike vesicles, 24.5 ± 4.0 nm diameter for smaller vesicles and 133.4 ± 63.3 nm diameter for larger vesicles (Fig. S9D/E). Finally, bilayer thickness was estimated from cryo-EM images as 4.03 ± 1.66 nm (*n* = 80, error = ±1 sd). Our measured value is within range of the pure DOPC bilayer thickness (5.5 ± 0.1 nm) measured by Leonenko *et al.*, determined using neutron scattering. This suggests ethanol could cause bilayer thinning, but higher accuracy measurements are needed to verify this.^64^ The variety of structures infers non-uniform lipid distribution between structures, as wormlike structures could be indicative of high localized DSPE-PEG_2000_ concentration within the highly curved ends. The combination of non-homogeneous DSPE-PEG_2000_ and ethanol lateral expansion has been thought to favour wormlike structures.^53^ The high curvature of vesicles under 100 nm in diameter may drive fusion into larger structures. Post VFF this could likely be avoided using higher PEGylated lipid fractions (>10 mol%) at the cost of moving from clinically relevant formulations.^65^

Next, CL + M was characterized by cryo-TEM images which were comprised of predominantly small vesicles (<100 nm) and a low number of larger vesicles (≈200 nm), with some exhibiting multilamellar features (Fig. 4F/G). Generally, menadione encapsulation appeared to lower the formation of wormlike vesicles (Fig. S9B and S11). This was confirmed using particle classification analysis approach as above. In contrast to CL − M, upon inclusion of menadione almost all particles were classified as smaller vesicles (95%), with small amounts of larger (2%) and wormlike vesicles (3%) present (Fig. S9C). Estimation of particle sizes confirms similar dimensions for CL + M as with CL − M: wormlike vesicles possessed dimensions of 76.1 ± 46.3 nm length × 16.3 ± 2.4 nm width, smaller vesicles 20.7 ± 4.4 nm diameter and larger vesicles 177.3 ± 54.0 nm diameter (Fig. S9D/E). This indicates that the presence of menadione has a significant impact on final particle morphology, reducing the number of wormlike vesicles, but does not appear to change the size of the three particle types observed. Bilayer thickness was estimated from the image as 3.91 ± 2.01 nm (*n* = 80, error = ±1 sd). This value is comparable to CL − M bilayer thickness, implying inclusion of menadione has an insignificant effect on bilayer thickness. Nevertheless, the high concentration of menadione in the membrane (70 μM, 18.91 mol%) may act to ease packing frustration and potentially reduce the formation of wormlike vesicles. Using X-ray diffraction, the lipid phase of the CL formulation was determined to be the L_α_ phase (Fig. S12/13). Based on the lipid phase, menadione could have a thinning effect comparable to ethanol bilayer lateral expansion,^53^ however this cannot be confirmed here due to measurement error. The presence of varied structures demonstrates the potential for VFF application to wormlike and multilamellar synthesis, through control of lipid formulation and flow-based parameters. Overall, cryo-TEM indicated the DLS by intensity plots were not fully representative of sample diversity.

**Fig. 5:**
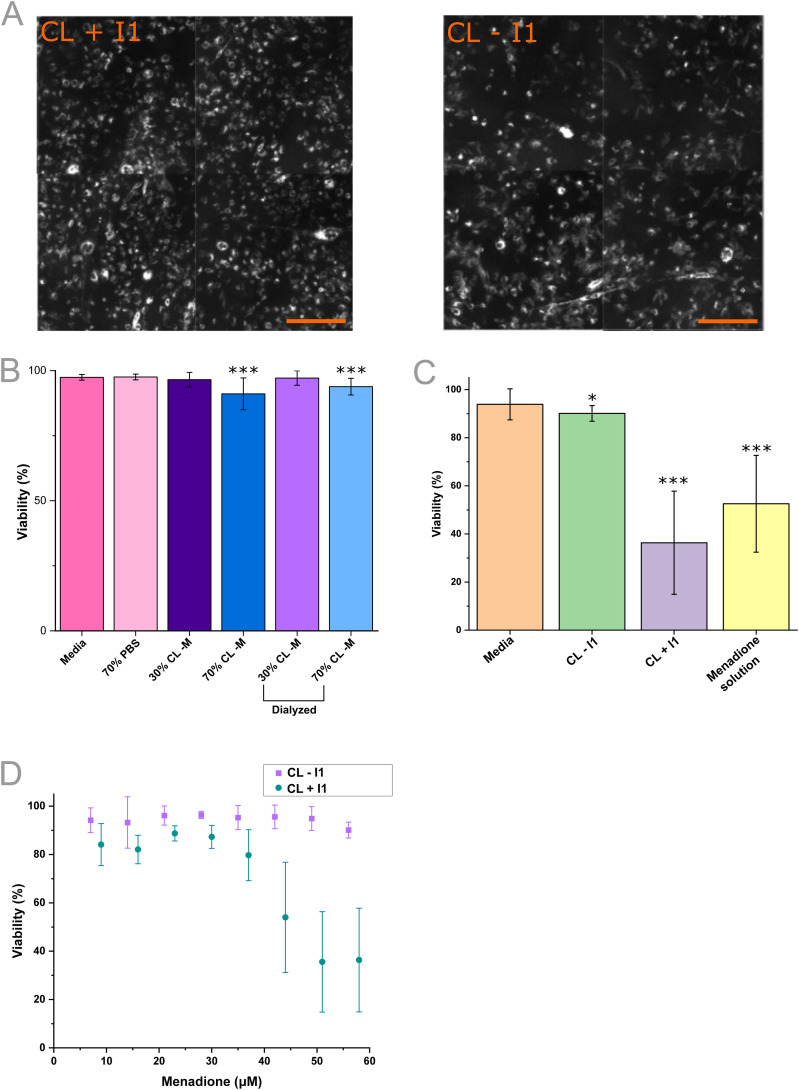
Application of VFF-produced vesicle nanocarriers to H9C2 cell culture. A) Accumulation of Texas-red DHPE (1 mol%) tagged vesicles with I1 targeting (left) (CL + I1, DOPC : cholesterol : DSPE-PEG_2000_ : DSPE-PEG_5000_-peptide 46 : 46 : 5 : 1 mol%) and CL formulation (right) (CL − I1, DOPC : cholesterol : DSPE-PEG_2000_ 46 : 46 : 6 mol%) in H9C2 cells after a 12 hour incubation period. Scale bars = 0.25 mm. B) Viability impact on H9C2 cultures treated with H9C2 media, 70 : 30 PBS : media, 30 : 70 and 70 : 30 vol% CL : media both undialyzed and dialyzed with a 3-hour incubation period (control *n* = 18, sample *n* = 36, from 3 biological replicates, error = ±1 sd). C) Viability change with the treatment of H9C2 culture with media, CL − I1 and CL + I1 liposomes containing 63 μM menadione and a 70 μM menadione solution over a 3-hour incubation (*n* = 18, 3 biological repeats, error = ±1 sd). D) Impact on viability determined from 3-hour incubation of increasing fractions of vesicle nanocarriers containing 70 μM menadione (15 ≥ *n* ≥ 11 from 3 biological repeats) (p* ≤ 0.05, p** ≤ 0.01, p*** ≤ 0.001 from a Student's *t*-test).

To address this the samples were additionally characterized by multi-angle DLS (MADLS) sizing by volume. MADLS sizing of the CL − M detected three separate populations within Fig. 5D. The first and most common was comprised of 35 nm structures, which are likely the small unilamellar liposomes observed in cryo-TEM. Particle distributions around 122 nm and 430 nm are likely the wormlike and/or larger vesicle particles observed within the sample respectively. This correlates with the number of particle types observed *via* cryo-EM (Fig. S9), with size differences between the largest peak in MADLS (430 nm) and cryo-EM (133.4 ± 63.3 nm) attributed to the small number of larger vesicles observable *via* cryo-EM in contrast to the population-level measurements obtained *via* MADLS. In contrast, the CL + M formulation exhibited a monomodal distribution centered around 75 nm. As cryo-TEM analysis showed 95% of measured particles being classified as smaller vesicles (Fig. S9C, 20.7 ± 4.4 nm diameter), this monomodal distribution is attributed to this smaller vesicle population. Again, cryo-EM measurements are ∼3.5× smaller than the equivalent MADLS measurement (75 *vs.* 20.7 nm), potentially indicating a systematic overestimation in particle size using MADLS compared to cryo-TEM imaging. As larger particle populations were not observed in MADLS, the small number of wormlike and larger vesicles observed in CL + M cryo-TEM analysis may accurately represent the morphology ratio present in the larger sample. Through comparison of MADLS and cryo-TEM, it is likely menadione encapsulation was successful and did not affect the bilayer phase of the membrane, despite reported membrane activity.^22,23^ Furthermore, the disordering effect of hydrophobic drug inclusion within VFF may reduce the heterogeneity of the resulting vesicles, improving sample quality.

To further study the inclusion of menadione, the UV-vis spectra of menadione-containing vesicles (47 : 46 : 7 DOPC : CHOL : DSPE-PEG_2000_, 1 mg mL^−1^, 1.36 mM) were taken (Fig. S14). A high concentration of menadione (0.4 mg mL^−1^, 2.32 mM) was utilized in the study to remain within the detection range of UV-vis which meant the sample is not fully representative of the previous encapsulation concentration. Encapsulation efficiency was estimated as 34.03 ± 0.23% (0.79 mM) of the 2.32 mM menadione (eqn (S2) and (S3)). This indicates that menadione loading in the vesicles is occurring at a 3 : 5 drug : lipid molar ratio (0.79 mM : 1.36 mM). Using this method provides an approximation to the encapsulation efficiency, as the menadione concentration was significantly higher (2.32 mM) than the concentration used in the DLS study (0.07 mM). As there is an excess of menadione in this encapsulation experiment (∼2 : 1 initial molar ratio of drug : lipid), it is likely that the lipid leaflet is fully saturated. In the other characterisation experiments (Fig. 4) menadione is included at an initial 1 : 5 drug : lipid ratio, implying that the vesicles could facilitate complete inclusion (100% encapsulation efficiency) of highly hydrophobic menadione. Such high encapsulation efficiency values have been recorded for similarly hydrophobic drugs such as griseofulvin when loaded into lipid vesicles from solvent phases.^69^

### 2.3. Application of myocardiocyte-targeting vesicle nanocarriers

After evaluating the VFF device for the production of vesicle drug delivery formulations, we next addressed the potential of these structures for targeted delivery (CL + I1, 47 : 46 : 5 : 1 : 19, DOPC : CHOL : DSPE-PEG_2000_ : DSPE-PEG_5000_-I1 : menadione mol%). In these experiments, the characterisation of the vesicle specificity was next evaluated, with a targeting element provided by the DSPE-PEG_5000_ conjugated I1 peptide (1 mol%).^16^ The I1 peptide was first identified using bio-panning and has been demonstrated to accumulate within the infarct-border zone within mice (as determined by heart : liver accumulation). It has also been studied with human RL-14 left ventricular cells, demonstrating its suitability for use here as a model targeting vector to cardiac cells.^16,66^ In this work, the potential interaction of the synthesized vesicles was first visualized by adding DHPE-Texas red (3 mol%) to each formulation, during VFF. The accumulation of CL − I1 and CL + I1 vesicles were explored by incubation with H9C2 cell culture for 12 hours before subsequent washing (to remove weakly bound particles) and fluorescence microscopy imaging (Fig. 5A). After the incubation, DHPE-Texas red staining was observed in all samples which indicated potential binding and/or internalization into H9C2 cells. CL + I1 and CL − I1 exhibited similar intensities, this implied both were accumulating at and/or internalized by H9C2 cells. DHPE-Texas red accumulation signified that there may be a non-specific interaction with all synthesized vesicles. To explore this further the impact on cell viability was explored for the CL formulation.

Increasing vesicle volume fractions without menadione (CL − M) were added to evaluate toxicity within H9C2 cell culture over 3 hours, using a viability assay (Fig. 5B).^67^ The viability assay employed Hoechst nuclei stain for the total cell population and DRAQ7 as a dead cell nuclei stain, with the respective counts used to determine cell viability (Fig. S15). Control samples were comprised of media and diluted media (30 : 70 media : PBS vol%). Over the 3-hour time course, these treatments showed low cell death (97.4 ± 1.1%, 97.6 ± 1.1% viability respectively). At 30 vol% CL − M (89.48 μM lipid), low cell death (96.5 ± 2.8%) was observed and at the higher 70 vol% CL − M (208.79 μM lipid) viability remained within experimental error (91.1 ± 6.1%). The higher volume fraction was expected to decrease viability, because of the residual ethanol within the sample (6.3 vol%, 1.09 M), a byproduct of the VFF process.

To address the residual ethanol toxicity, the samples were dialyzed and applied to the H9C2 culture in parallel. In the dialyzed CL − M samples, 30 vol% yielded minimal cell death (97.2 ± 2.8%) and at 70 vol% viability was distinguishably lower (93.8 ± 3.2%). Dialyzed 30 vol% samples remained within experimental error of the untreated samples, although a subtle decrease in toxicity was observed in the 70 vol% samples. Ethanol was thought to be largely removed by dialysis (12 hours, Slide-A-lyzer™ Mini), though we expect some to be retained within the bilayer (as indicated by Fig. 3D), marking a potential limitation of passive flow-based vesicle production when studying for delivery applications.^53^ Within this study, the impact on viability was considered low (<10%) within the 3-hour time frame.

To establish whether vesicles containing the I1 targeting peptide increased the efficacy of menadione delivery *versus* a non-targeting formulation, the viability assay was again employed (Fig. 5C).^67^ Vesicles ± I1 targeting peptide was loaded with hydrophobic menadione during the VFF synthesis, and vesicles at 268.45 μM lipid concentration loaded with 63 μM menadione (CL − I1) resulted in comparable viability (90.1 ± 3.3%) compared to the media control (93.9 ± 6.4%). When treated with I1 targeting vesicles (CL + I1) at 268.45 μM lipid concentration prepared with 63 μM menadione, viability significantly decreased (36.3 ± 21.4%) to a level comparable with treatment with a 70 μM menadione solution (52.5 ± 20.1%). From the viability set it is clear that inclusion of the I1 peptide facilitated the delivery of menadione, although the route of delivery and specificity of the entity is unclear.^16^ This data set indicated that VFF devices can be used to produce targeted vesicle nanocarriers that exhibit a differential effect on cells *in vitro*, as demonstrated through the viability assay.

To further characterize vesicle efficacy, the dose-dependent behaviour was explored within H9C2 culture (Fig. 5D). The vesicle dosing was increased such that both the vesicle concentration and menadione concentration increased. Increasing CL − I1 dosage resulted in a negligible decrease in viability (94.20 ± 5.10%, to 90.11 ± 3.27%) within the explored concentration range and 3-hour incubation, indicating that vesicles lacking the I1 targeting peptide possessed negligible toxicity to H9C2 cells. Increasing the dosage of CL + I1 vesicles resulted in a significant decrease in viability (36.3 ± 21.4%) beyond an estimated 35 μM menadione dose. The EC_50_ of the +I1 vesicles was determined to occur at 198.27 μM lipid concentration, containing 51.7 μM menadione. This highlights the applicability of VFF to the screening of nanocarriers such as vesicles for drug delivery.

## 3. Conclusion

In summary, the ability to produce vesicle-based nanoparticle formulations within a cleanroom-free fabricated PMMA device was first presented. The low cost and high processability of PMMA allows us to adopt a laser-cutting approach, which is maximally accessible compared to previous VFF devices which have required the use of machine milling, hot embossing or deep reactive ion etching. In this work we used the multilayer VFF device to produce a variety of lipid vesicle-based structures, from single DOPC vesicles to quaternary compositions capable of targeting H9C2 cardiac cells. Comparing our device against existing VFF technologies, we note that our device possesses similar maximum TFR to layer-by-layer devices assembled by Hood *et al.*^68^ (2.1 *vs.* 4.5 ml min^−1^), similar aspect ratios to previous devices by the Devoe group (50 *vs.* 100 (ref. 68) and 40 (ref. 52) respectively), and competitive production rates compared to COC and DLP VFF chips (105 *vs.* 90 (ref. 68) and 235 (ref. 52) mg per hour lipid respectively, Table S4). This indicates that our devices are accessible and capable of producing lipid nanocarriers similar to existing VFF technologies. By applying the operating parameters set out in this work, our PMMA device could be utilized for the synthesis of increasingly biomimetic particles such as synthetic exosomes.^69^ The VFF device was effective in producing DOPC nanocarriers with high levels of size control, with the greatest difference occurring between FRRs 1.5–4 (185.8 ± 21.6 nm–98.75 ± 18.0 nm), and a gradual reduction in size observed between FRR 4–25 (particle diameter of 81.53 nm ± 14.96 nm obtained for FRR 25, TFR = 2.6 mL min^−1^). By incorporating the BC10 molecular rotor, the effect of residual ethanol within the lipid bilayer on microviscosity was further quantified, a first assessment of a potential limitation of MHF/VFF. The microviscosity data suggested that even at FRR > 5, membrane microviscosity is reduced compared to a solvent-free sonicated vesicle control, indicating that residual ethanol solvent is retained in the nanocarrier membranes. However, successful dialysis was achieved for the FRR 5 samples after 12 hours, again demonstrated *via* microviscosity measurements. VFF-produced liposomes exhibited lower variation in their reported microviscosity suggesting VFF provides more monodisperse microviscosity within samples. We expect that, given that hydrated vesicle samples are typically highly multilamellar, the range in reported microviscosity may represent the different curvatures of the multilamellar lipid bilayers.^70^ In the latter work, FRR 10 and TFR = 1.1 mL min^−1^ were applied as this provided a lower perturbation of the bilayer by ethanol and a higher concentration of resultant liposomes when compared with FRR 25 for a given lipid in ethanol concentration. Increasing FRR results in a decrease in lipid concentration and relation, lipid vesicle concentration.

Next, the VFF design employed a multi-component formulation (47 : 46 : 7, DOPC : cholesterol : DSPE-PEG_2000_ mol%) demonstrating the translation of VFF to more complex vesicle nanocarriers. DLS sizing by intensity was found to misrepresent sample quality and a combination of cryo-TEM and MADLS size by volume was used to characterise the vesicles. Samples were found to be predominantly small unilamellar liposomes (<100 nm diameter), with a lower presence of both worm-like vesicles (200–400 nm length) and multilamellar liposomes (200 nm diameter). When encapsulating menadione (18.94 mol%) liposomal swelling was observed by DLS and a lower incidence of worm-like vesicle structures was observed, with 95% of particles of a spherical vesicle morphology indicating menadione encapsulation may reduce sample dispersity. The cryo-TEM data demonstrated the VFF method produces predominantly singly lamellar particles which could present a significant improvement upon the extrusion method.

To further understand menadione encapsulation a UV-visible study was employed to estimate encapsulation efficiency, using a higher menadione concentration (2.32 mM). Encapsulation was estimated as 34.03 ± 0.23% (0.79 mM) using menadione standard solutions where menadione was in excess of the total lipid concentration. For all other experiments, menadione was loaded as 19 mol% of the lipid film, which implies that the vesicles could facilitate complete inclusion of the highly hydrophobic menadione drug. Overall, characterisation studies gave promising evidence that menadione was successfully encapsulated in one step on the device into vesicular nanocarriers.

The addition of DHPE-Texas red liposomes to the H9C2 cell culture was applied to visualise potential nanocarrier–cell interactions. Incubation over 12 hours appear to show accumulation of both targeting (CL + I1) and non-targeting (CL − I1) vesicles at the H9C2 cells. Next, a viability assay was employed to evaluate the toxicity of vesicles (CL − I1, DOPC : cholesterol : DSPE-PEG_2000_ 46 : 46 : 6 mol%) produced by VFF. The vesicles were found to be minimally toxic (>90% viability) at even high fractions (70 vol%) and residual ethanol within the sample exhibited a low impact on viability (91.1 ± 6.1%). Vesicles encapsulating menadione were then explored, to establish whether incorporation of the targeting peptide I1 could facilitate the delivery of menadione. When added to H9C2 cells, convincing evidence for targeting was provided by the decrease in viability for I1 targeting vesicles (CL + I1, DOPC : cholesterol : DSPE-PEG_2000_ : DSPE-PEG_5000_-peptide 46 : 46 : 5 : 1 mol%). The inhibited viability using CL + I1 (36.3 ± 21.4%) far exceeds the inhibition by the non-targeting formulation CL − I1 (90.1 ± 3.3%). The difference from the targeting effect was further characterised by the titration of CL + I1 vesicles containing menadione in H9C2 culture. Clinical opportunities for cardiomyocytes treatment include the delivery of genetic cargo to modify intracellular gene expression^71^ and the delivery of compounds for myocardial regeneration.^72^ Here, we show the I1-mediated delivery of menadione using vesicle nanocarriers produced from PMMA VFF devices, acting as proof of concept for the production of targeted vesicles from our device as a prospective nanomedicine formulation.

In conclusion, this work demonstrates the full cycle of therapeutic vesicle nanocarrier synthesis, characterisation, and application, outlining an affordable, cleanroom-free millifluidic platform for the synthesis of bioinspired vesicle nanocarriers, including nanoscale artificial cell development.^73^ This process could be used to rapidly screen nanocarrier formulations by on-chip mixing and reasonable throughput (up to 2.6 mL min^−1^). By coupling, this flow-based production method with cell culture models of disease, the efficacy and delivery properties of these formulation libraries can be evaluated. This will accelerate the pace of vesicle-based drug delivery systems as well as the development of new nanoscale artificial cell models for application in fundamental biology as well as biomedical translation.

## 4. Experimental

Hazards for associated experimental methods can be found in the SI.

### VFF fabrication

PMMA layers described in Fig. 2A and S2 were cut from Clarex-001 (Weatherall Equipment and Instruments Ltd, UK) using a laser cutter (VLS 2.30, Universal Laser Systems, Austria). To bond, PMMA was layered onto a heating block comprised of two 90 × 50 × 15 mm aluminum blocks (width × length × depth) with threaded M6 holes in each corner. The base featured alignment pins that were 3 mm (OD), centered, and displaced by 60 mm, these were used to feed the PMMA pieces using the alignment holes featured in the design. As PMMA pieces were layered, 70 vol% ethanol (VWR) was sprayed in between layers to facilitate bonding.^50^ Next, M6 bolts were used to sandwich the PMMA layers between aluminum blocks using 1 N m^−1^ torque per M6 bolt. After leaving under pressure for 15 minutes, allowing excess ethanol to evaporate, the block was placed on a hotplate heated to 70 °C and left for 2 hours.

Microfluidic adapters were designed on Autodesk fusion 360 and printed in PET-G by an Ultimaker S3, 3D Prints were prepared using a CURA slicer with raft adhesion and a 50% gyroid pattern infill. Adhesion to the PMMA chip was achieved using quick-set epoxy (RS components) and alignment used PTFE (1/16″) capillaries (Darwin microfluidics) to guide adapters and prevent the glue from entering the VFF device.

### VFF DOPC sample preparation

The ethanol phase was prepared by first aliquoting a DOPC in chloroform solution (Avanti, 25 mg mL^−1^) into a glass vial. The lipid in the chloroform solution was then dried onto the vial using a nitrogen stream, achieving a thin film. Following drying, the sample was lyophilised for 12 hours to remove residual chloroform. After lyophilising the film was dissolved in anhydrous ethanol (VWR) to achieve the desired lipid concentration (5.0 mg mL^−1^) and mixed using vortexing (1500 rpm, 90 seconds). For the buffer phase in VFF, filtered phosphate-buffered saline (VWR) was employed.

### VFF setup

For perfusion, syringe pumps were loaded with syringes (Henke-Ject), for the ethanol and buffer solutions. To connect syringes, poly(tetrafluoroethylene) (PTFE) tubing (0.5 mm ID and 1/16″ OD) was used in conjunction with Luer lock to 1/16″ head fitting F/F adapters, polyether ether ketone (PEEK) 1/16″ head fittings and PTFE 1/16″ ferrules (Fisher Scientific Ltd.). PTFE thread tape (RS components) was applied to the head fitting thread to improve the seal at the poly(ethylene)terephthalate glycol (PET-G, Ultimaker) adapter. The VFF device was secured upright in the 3D-printed PET-G mount and purged with filtered phosphate-buffered saline to remove trapped air before usage. All 3D prints were achieved using an Ultimaker S3.

### FRR and TFR sweep

In the FRR sweep the ethanol flow rate was fixed at 0.1 mL min^−1^ and the total aqueous flow rate was increased (0.1–2.5 mL min^−1^) to increase both FRR (1–25) and TFR (0.2–2.6 mL min^−1^). For the TFR sweep, FRR was fixed at 5 and TFR was increased (0.3–2.1 mL min^−1^). All samples were run through the device for a minimum of two minutes before sample collection, this ensured a steady flow rate was achieved.

### BODIPY rotor microviscosity measurement

Rotor incorporation was achieved by the addition of the well-characterised BODIPY-C10 (BC10) dye in chloroform to the lipid in the chloroform solution.^56^ Rotor concentration was chosen to achieve a final sample concentration of 0.5 mol%, post flow focusing, to avoid aggregation-induced quenching of the fluorescent probe. The chloroform solution was then prepared using the VFF sample procedure and applied within VFF using the previously described procedure. Dialysis was performed on one large-batch VFF sample which was then split into multiple dialysis cups to make replicates. Buffer was replaced at the following time points: 5, 12, 22 and 29 hours. For the sonicated sample, the film was hydrated in PBS and tip sonicated (1 s on, 3 s off) for ∼45 minutes at room temperature.

For fluorescence lifetime measurements, the liposome samples were placed in a 1 mL volume quartz cuvette (10 mm path length) and the temperature was set to 22 ± 1 °C. A 404 nm pulsed laser (NanoLED) was used for excitation, and the time-resolved fluorescence decay traces were recorded at 515 nm using a time-correlated single photon counting (TCSPC) instrument, Horiba Jobin Yvon IBH5000 F. Decay traces were fitted using DAS® software, and the obtained lifetime was used to calculate the microviscosity according to a previously reported calibration:^73^log_10_ *τ* = 0.4569 log_10_ *η* − 0.75614Once fitted, box plots display the 25–75% range, error bars represent ± S.D., the median is shown by a horizontal line and the mean by a line.

### VFF CL sample preparation

The ethanol phase was prepared by first aliquoting a DOPC (Avanti, 25 mg mL^−1^), cholesterol (Sigma Aldrich, prepared to 25 mg mL^−1^), and DSPE-PEG_2000_ (Avanti, 25 mg mL^−1^) in chloroform solutions into a glass vial (47 : 46 : 7, DOPC : CHOL : DSPE-PEG_2000_ mol%). The lipid in the chloroform solution was then dried onto the vial using a nitrogen stream, achieving a thin film. Following drying, the sample was lyophilised for 12 hours to remove residual chloroform. After lyophilising the film was dissolved in anhydrous ethanol (VWR) to achieve the desired concentration (10 mg ml^−1^) and mixed using vortexing (1500 rpm, 90 seconds). For the buffer phase in VFF, filtered phosphate-buffered saline (VWR) was employed. For menadione samples, a menadione (Sigma Aldrich) in chloroform stock (4 mg mL^−1^) was prepared and added before drying (47 : 46 : 7 : 19, DOPC : CHOL : DSPE-PEG_2000_ : menadione mol%). Samples were produced at FRR 10 and TFR 1.1 mL min^−1^ within the VFF device.

### Ultraviolet-visible study

Lipid vesicle (47 : 46 : 7, DOPC : CHOL : DSPE-PEG_2000_ mol%) samples were produced with a 10 mg mL^−1^ lipid in an ethanol solution containing a higher menadione concentration (4 mg mL^−1^). Samples were produced at FRR 10 and TFR 1.1 mL min^−1^ within the VFF device. When collected a biochrom UV-vis spectrophotometer was used to read the absorption from 200–400 nm for the 1 mL samples, in cuvettes (Kartell). Menadione standards were made by dissolving menadione films in 1 mL of PBS buffer, ultrasound was used to mix standard solutions before analysis.

### DLS and MADLS sizing

DLS samples were measured using a Malvern Zetasizer Ultra (ZSU5700) and polystyrene sample cuvettes (Kartell). Post flow focusing, samples were diluted 1 : 4 in PBS buffer, and measurements were taken at 20 °C following a 2-minute temperature equilibration. A minimum of 5 rounds of 20 measurement runs were applied for each sample. Size average and polydispersity index for each sample was estimated by Malvern Zetasizer software. For MADLS, a DLS size scan was first used to determine the average derived count rate (kcps, typically 200–400), this value was then applied within the MADLS scan. MADLS measurements consisted of 5 rounds of 20 measurements for each scattering type (forward, back, side).

### Cryogenic transmission electron microscopy

Cryo-electron microscopy samples were undertaken at the Imperial College Centre for Structural Biology and were taken on an FEI Tecnai 12 bio twin 120 kV using a TVIPS XF416 4K CMOS detector. For imaging, 3 μL samples were transferred onto a carbon grid (Quantifoil™ R 2/2 on 300 copper mesh; Jena Bioscience), in a Vitrobot (Thermo Fisher) at 90% humidity. Sample grids were blotted (blot time: 5 s; blot force: −1; wait time: 35 s) and then immediately plunged into a liquid ethane bath contained within a liquid nitrogen reservoir. The grid was transferred to a grid holder at −180 °C. Grids were transferred under liquid nitrogen to an electron microscope sample holder (Cryo Transfer Tomography holder, Eden Instruments; Model 2550). Defocus values used were between −0.5 and −3 μM, depending on the chosen magnification which ranged from 26 000–52 000×.

### H9C2 culture

H9C2 rat cardiomyocytes (ATCC) were plated (10 000 cells per cm^2^ in tissue culture-treated T75 Falcon flasks). Specific cell concentrations were measured using a Beckman Coulter VicellXR cell counter. Cells were pelleted (Eppendorf 5810R centrifuge at 4 °C, 300RCF, for 3 minutes) and resuspended in media was used to achieve specific seeding concentrations. For cell culturing medium, DMEM (ATCC high glucose DMEM) supplemented with 10 vol% FBS (Gibco), 1 vol% l-glutamine (Gibco, 10 mM), and 1 vol% antibiotic–antimycotic (Gibco, 100×) was used. Cells were grown in a Thermo Scientific incubator (37 °C, 5% CO_2_). Replating was achieved using 250 μL trypsin solution (Gibco, 0.25%) in EDTA and quenching with cell culture medium.

### Texas red accumulation

Lipid vesicles were generated using the VFF procedure at FRR 10 and TFR 1.1 mL min^−1^, applying 1 mol% DHPE-Texas red within the ethanol solution. Before cell application, vesicles were sterilised through a 0.2 μm syringe filter. H9C2 cells were plated at 10 000 cells per well in a Greiner half area 96 well plate, cells were left for 12 hours to achieve >80% confluence before the study. Texas red tagged vesicles were added at 50 vol% in culturing media and left for 12 hours before aspiration, washing with two 50 μL PBS fractions. After washing, staining with 10 μL of a Hoechst 33342 (50 μg mL^−1^, Thermo Fisher) solution in DMEM for 20 minutes was undertaken followed by the replacement of culturing media. Images were taken using were analysed on a Cellomics™ ArrayScan VTI, (10× magnification), with two fluorescent channels for Hoechst 33342 and Texas red respectively.

### Viability assay

Well plates were prepared analogously to the Texas red assay and vesicles were generated at identical VFF conditions, with a final concentration of 268 μM lipid and 70 μM menadione. Cells were left 12 hours to reach >80% confluence, confluence was measured by a Cloneselect imager. Following incubation at each condition, cells were first stained using 10 μL of a DRAQ7 (0.03 mM, Biolegend) solution, for 20 minutes. Wells were then aspirated and stained with 10 μL of a Hoechst 33342 (50 μg mL^−1^, Thermo Fisher) solution in DMEM for 20 minutes, the aspirated and replaced by media. Images were taken using were analysed on a Cellomics™ ArrayScan (Thermo Fisher) with 10× magnification and two fluorescent channels for Hoechst 33342 and DRAQ7 respectively (Fig. S12). Analysis was undertaken using HCS Studio™ Cell Analysis Software, and viability was assessed from the ratio of Hoechst-positive cells (Both live & dead cells) to that of Hoechst and DRAQ7-positive (Dead cells) cells:
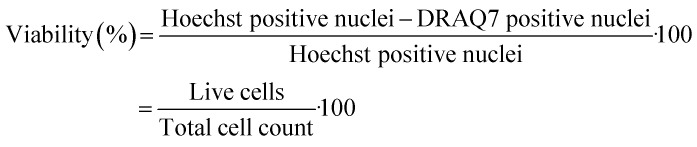


### Statistical analysis

For reporting and analysing membrane microviscosity measurements, a one-way ANOVA, Tukey *post hoc* test was applied using Origin 2024. Probability (*p*) values were expressed as the following: ns = *p* > 0.05, * = 0.05 > *p* > 0.01, ** = 0.1 > *p* > 0.01, *** = *p* < 0.01.

For reporting and analysing cell viability plots, a one-sided Students *t*-test was applied, using Origin 2024. Probability (*p*) values were expressed as the following p* ≤ 0.05, p** ≤ 0.01, p*** ≤ 0.001. Viability data was achieved from *n* = 18 samples comprised of 3 biological repeats and error is plotted at ±1 standard deviation.

## Author contributions

MN, JWH and OC conceived and supervised the study. CDH, SM, CPP and MPP designed and performed the experiments, and analysed experimental data. KAK provided the synthesised I1 peptide. CDH, JWH and OC wrote the manuscript. CDH, CPP, MPP, KAK, MKK, YE, NJB, MN, JWH and OC revised the manuscript.

## Conflicts of interest

There are no conflicts of interest to declare.

## Supplementary Material

LC-026-D3LC00995E-s001

## Data Availability

Essential data are provided in the main text and the supplementary information (SI). Data is available from the corresponding authors upon reasonable request. Supplementary information containing associated experimental hazards, supplementary figures and tables is available. See DOI: https://doi.org/10.1039/d3lc00995e.
