# Supplementary material for: Affordable, cleanroom-free millifluidic production of targeted lipid nanocarriers via additive manufacturing
Source: Lab Chip. 2026 Jan 7;26(3):635–49. doi: 10.1039/d3lc00995e (PMC12784244; doi:10.1039/d3lc00995e)
Supplement: LC-026-D3LC00995E-s001 [file LC-026-D3LC00995E-s001.zip › lc-art-11-2023-000995 - supplementary information.pdf]

# Supporting Information

## Associated experimental hazards

**VFF fabrication:** Hazards include use of (contained) lasers, use of ethanol (highly flammable, serious eye irritation) and use of elevated temperatures (burn risk). Bonding step undertaken in a fume hood to prevent exposure to ethanol.

**VFF lipid and menadione sample preparation:** Hazards include use of menadione (harmful if swallowed, skin irritant, serious eye irritant, very toxic to aquatic life), use of chloroform (toxic if inhaled, suspected carcinogen) and use of ethanol (highly flammable, serious eye irritation). Samples prepared in a fume hood to prevent exposure to solvent.

**Cryogenic transmission electron microscopy:** Hazards include the use of liquid nitrogen (cold burn and asphyxiation) and liquid ethane (cold burn, asphyxiation, explosive). Thermal gloves, face covering, and an insulated apron is used when handling liquid nitrogen and ethane. Liquid ethane is handled in in a fume hood, and both chemicals are stored in specifically designated vessels.

**Cell culture:** Culturing hazards include the handling of animal cell lines. All animal cell lines are handled as an ACDP (Biosafety) Group 2 organism in case of contaminants. Cells are manipulated in a laminar flow hood with standard tissue culture PPE to prevent infection of cells or operator. Imaging hazards include the use of DRAQ7 (causes skin irritation, serious eye irritation, may cause serious respiratory irritation) and Hoechst 33342 (harmful if swallowed, causes skin irritation, suspected of causing genetic defects).

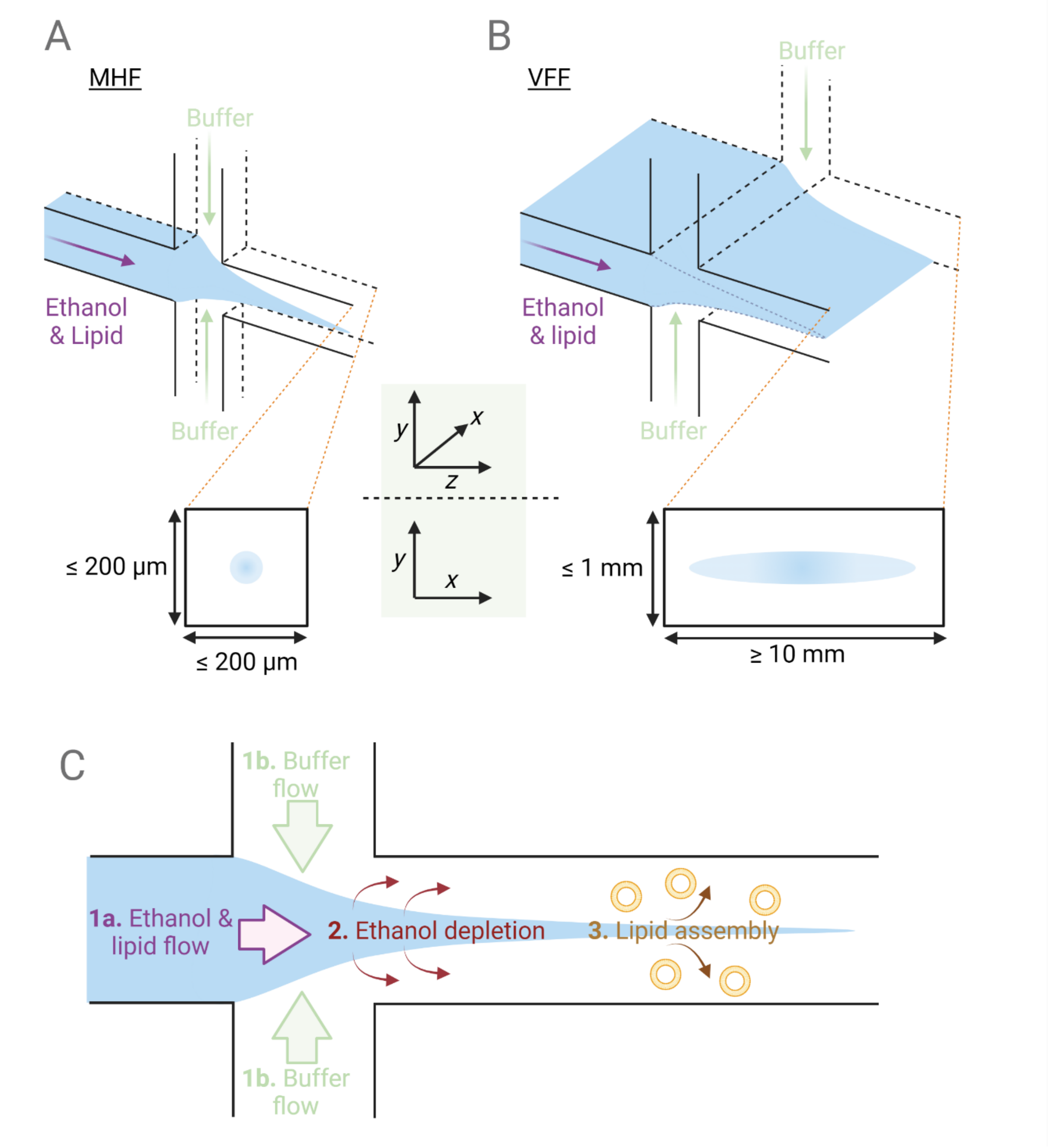


**Figure S1. Depiction of MHF and VFF. A)** Schematic of MHF process where a lipid in ethanol solution is co-flowed with aqueous buffer in a microfluidic cross-flow junction comprised of square channels (Channel cross-section of outlet channel shown below). **B)** Schematic of VFF process where a lipid in ethanol solution is co-flowed with buffer analogously to MHF though with a higher aspect ratio rectangular channel, resulting in a millifluidic device (Channel cross-section of outlet channel shown below). **C)** 2D representation of MHF mechanism where flow focusing of ethanol (1a) by buffer (1b) results in depletion of the ethanol stream (2) and subsequent self-assembly of lipid structures (3) through a purported disc-like micelle intermediate.

**Equation S1** Price of device calculation, based on UK valuations (December 2022).

Clarex-001 price per $mm^2$ (Weatherall): 680/(500 x 300) = 4.53 x $10^{-3}$ pence/$mm^2$

Clarex cost = Chip area x price per $mm^2$ = (72 x 36 x 7) x 4.53 x $10^{-3}$ = 82.25 pence

PET-G price per gram (RS components) = 4.46 pence $g^{-1}$

PET-G cost = Print mass x price per gram = 4 x 4.46 = 17.86 pence

Adhesive cost (RS components) = 30.20 pence $mL^{-1}$

Approximate adhesive cost = Adhesive volume x price per mL ≈ 30.2 pence

Approximate total material cost = Clarex cost + PET-G cost + Approx. adhesive cost = **£1.30**

*Tables of experimental flow rate conditions*

| FRR | Total ethanol flow rate (mL $min^{-1}$) | Total aqueous flow rate (mL $min^{-1}$) | TFR (mL $min^{-1}$) |
|---|---|---|---|
| **1** | 0.10 | 0.10 | 0.20 |
| **1.5** | 0.10 | 0.15 | 0.25 |
| **2** | 0.10 | 0.20 | 0.30 |
| **2.5** | 0.10 | 0.25 | 0.35 |
| **3** | 0.10 | 0.30 | 0.40 |
| **3.5** | 0.10 | 0.35 | 0.45 |
| **4** | 0.10 | 0.40 | 0.50 |
| **4.5** | 0.10 | 0.45 | 0.55 |
| **5** | 0.10 | 0.50 | 0.60 |
| **5.5** | 0.10 | 0.55 | 0.65 |
| **6** | 0.10 | 0.60 | 0.70 |
| **6.5** | 0.10 | 0.65 | 0.75 |
| **7** | 0.10 | 0.70 | 0.80 |
| **7.5** | 0.10 | 0.75 | 0.85 |
| **8** | 0.10 | 0.80 | 0.90 |
| **10** | 0.10 | 1.00 | 1.10 |
| **25** | 0.10 | 2.50 | 2.60 |

**Table S1.** FRR sweep applied values.

| **TFR ($mL\ min^{-1}$)** | **Total ethanol flow rate ($mL\ min^{-1}$)** | **Total aqueous flow rate ($mL\ min^{-1}$)** | **FRR** |
|---|---|---|---|
| **0.3** | 0.05 | 0.25 | 5 |
| **0.6** | 0.1 | 0.5 | 5 |
| **0.9** | 0.15 | 0.75 | 5 |
| **1.2** | 0.2 | 1.0 | 5 |
| **1.5** | 0.25 | 1.25 | 5 |
| **1.8** | 0.3 | 1.5 | 5 |
| **2.1** | 0.35 | 1.75 | 5 |

**Table S2.** TFR sweep applied values.

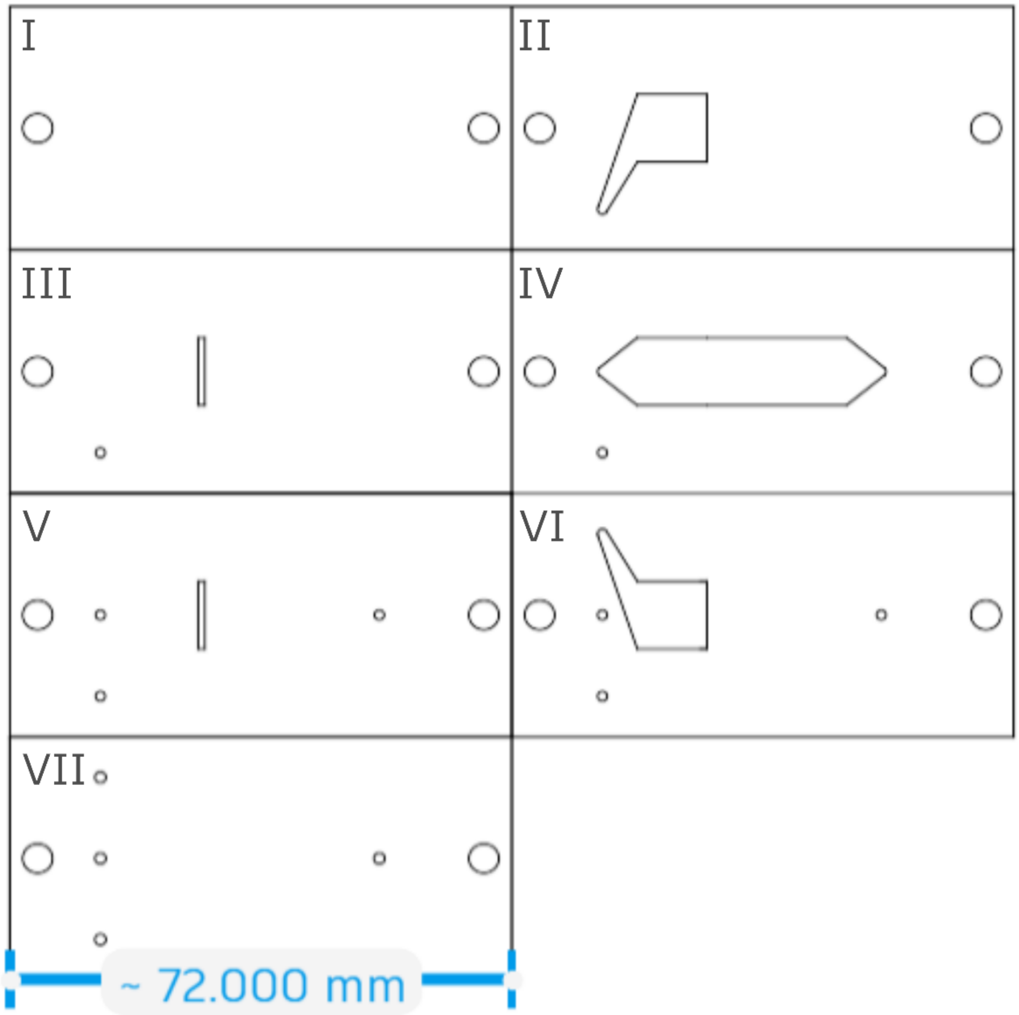


**Figure S2. Layer by layer schematic of VFF device.** Each layer of the VFF device is shown here, where each layer can be simultaneously printed from a single 1 mm sheet of PMMA**.** Part numbers are annotated on the image, corresponding to the part numbers listed in Figure 2A. The width of each part of the device is 72 mm. This schematic can be found as an accompanying file to this manuscript.

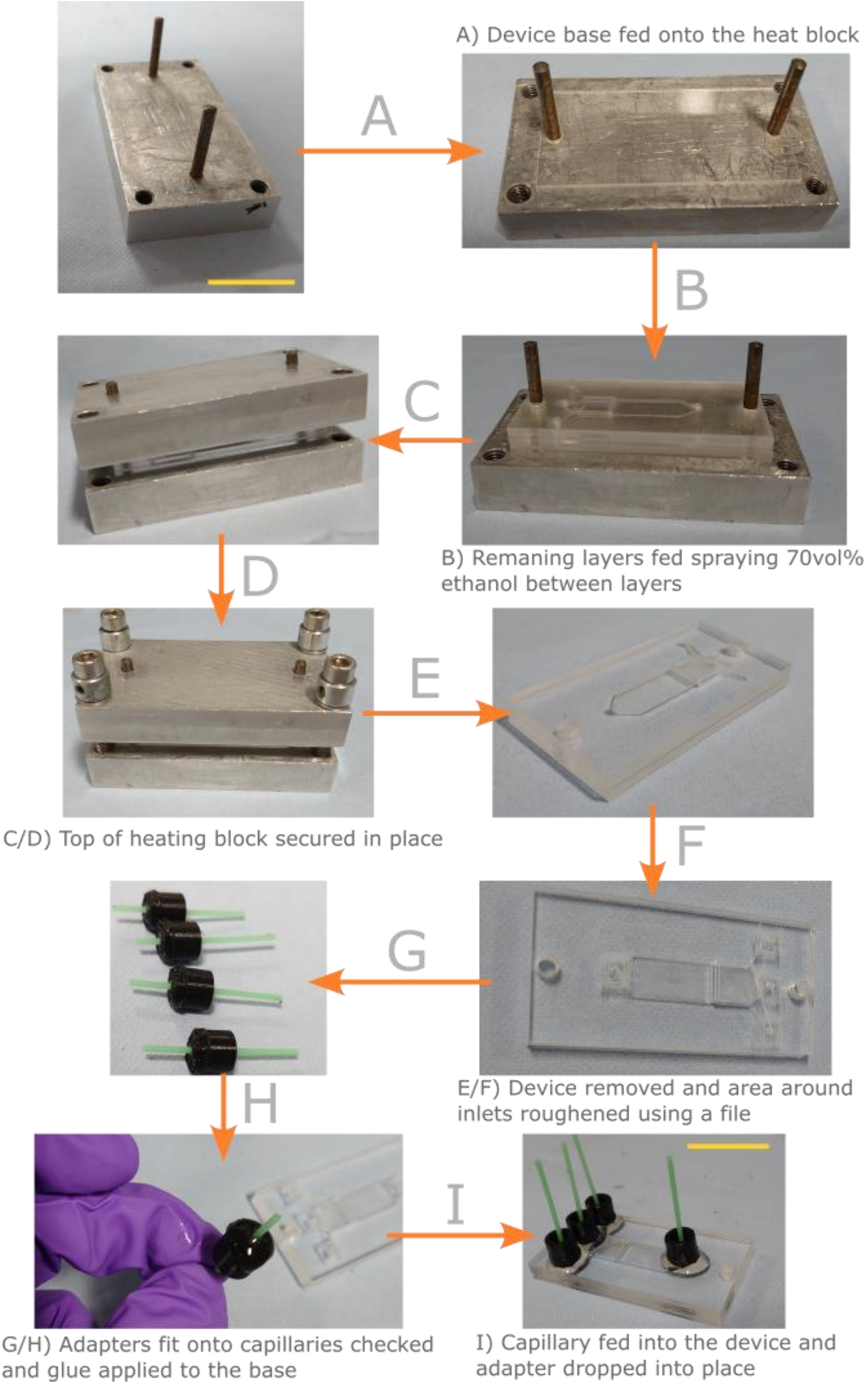
A
A) Device base fed onto the heat block
B
C
B) Remaning layers fed spraying 70vol% ethanol between layers
D
E
C/D) Top of heating block secured in place
F
G
E/F) Device removed and area around inlets roughened using a file
H
I
G/H) Adapters fit onto capillaries checked and glue applied to the base
I) Capillary fed into the device and adapter dropped into place

**Figure S3. Staged VFF device fabrication.** First showing the aluminum heat block (Top left, scale bar = 30 mm). A) The PMMA device base cut-out is fed onto the alignment pins of the heating block. B) The other PMMA layers are built up, spraying 70vol% ethanol in between layers as they are fed onto the heat block. C) The aluminum top of the heating block is fed on top of the design, sandwiching it in place. D) Four M6 bolts are used in each corner to secure the top in place and pressure is applied by setting the torque of each bolt to 1 $Nm^{-1}$ using a torque wrench. E) The block, containing the device, is heated at 70$^{o}$C on a hotplate for 120 minutes, and then the device is removed from the block. F) The areas around the device inlets are roughened using a file. G) The printed adapters (black) are checked to ensure the PTFE capillary (green) has a close fit. H) Epoxy resin is applied to the base of the adapter (black). I) Capillaries are fed into the inlet holes to align the adapters and the adapter is dropped into contact with the device, this is repeated until all inlets and outlets are adhered (Scale bar = 30 mm).

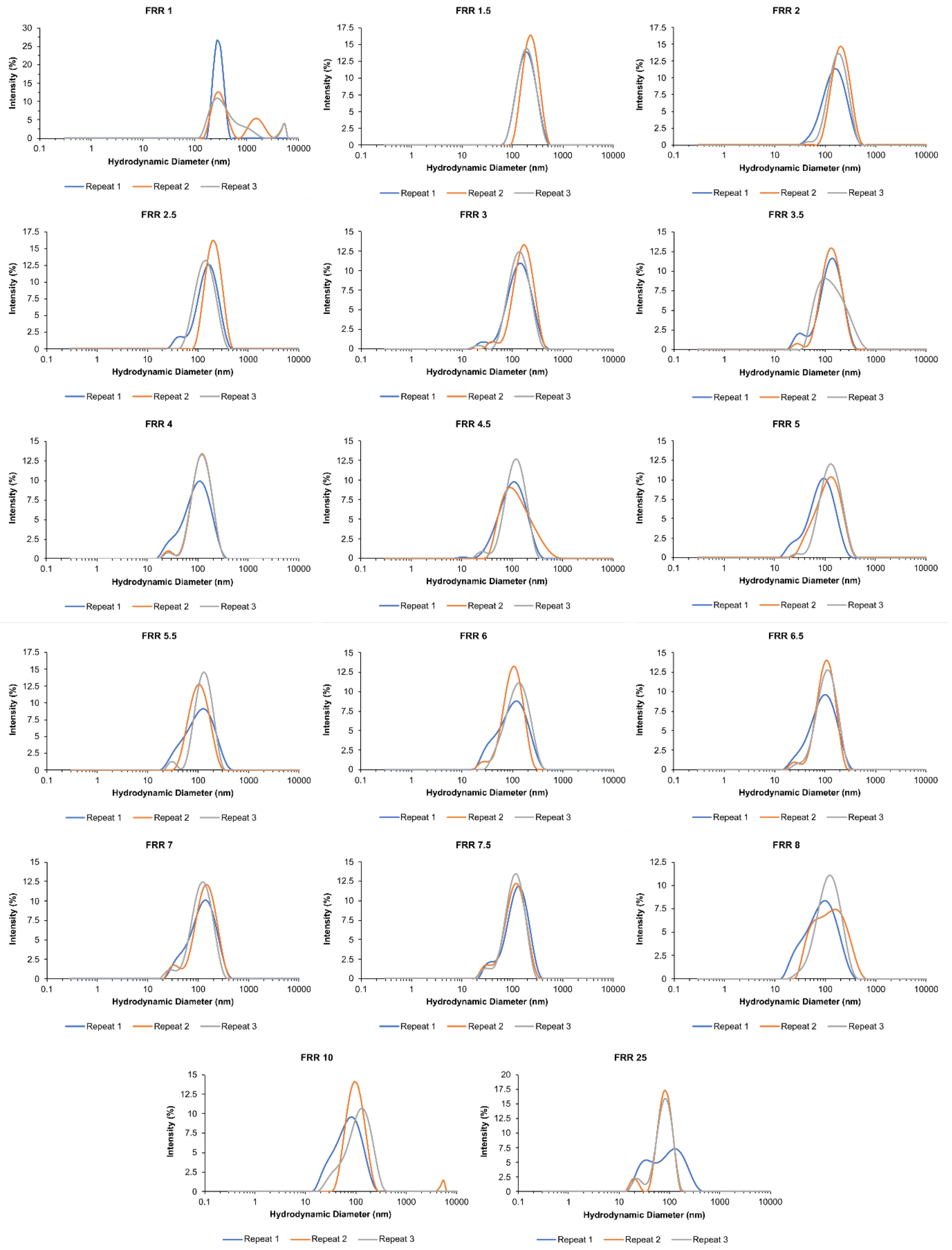


**Figure S4. Size distributions of DOPC nanocarriers produced via the PMMA VFF device at varying FRR (5.0 mgml$^{-1}$ DOPC lipids in ethanol and a PBS buffer phase).** Dynamic light scattering intensity size distributions are shown for each experimental repeat, FRR of each experiment is shown in the title of each graph. The

mean hydrodynamic diameter in Figure 3A is calculated from the hydrodynamic diameter of the most intense peak, which is ~100-200 nm in diameter, and in each case is attributed to vesicle nanocarriers.

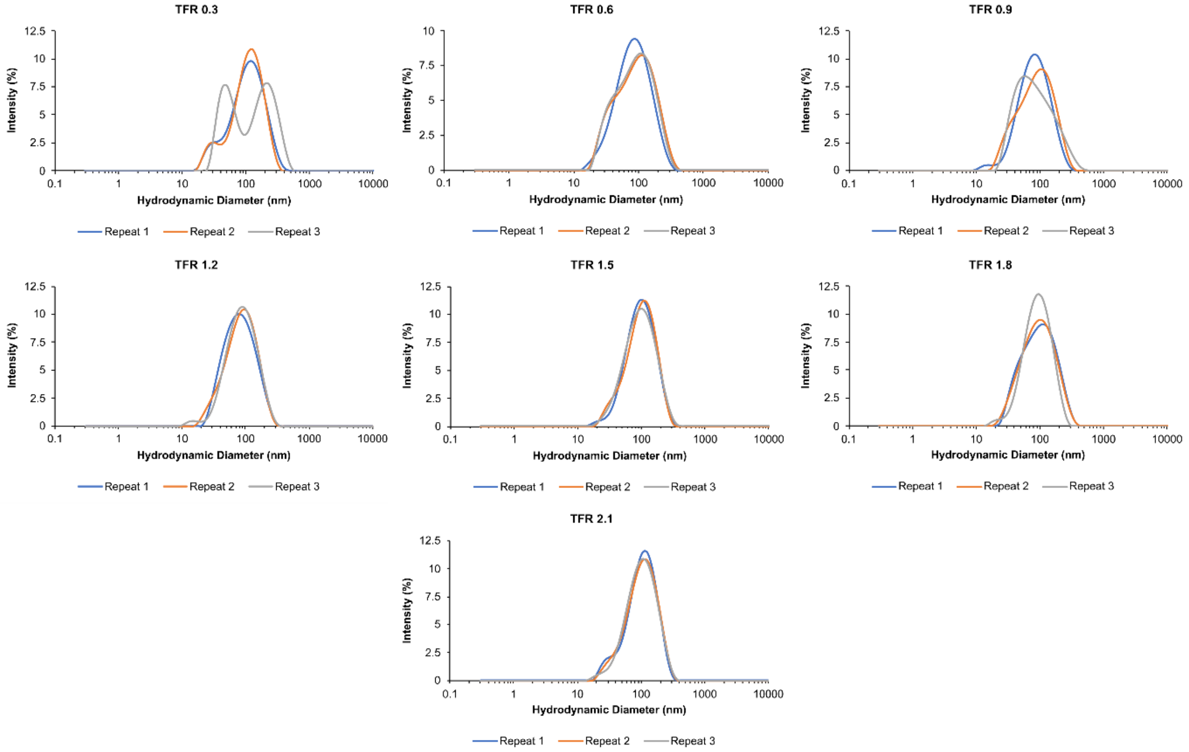


**Figure S5. Size distributions of DOPC nanocarriers produced via the PMMA device at varying TFR (5.0 mgml$^{-1}$ DOPC lipids in ethanol and a PBS buffer phase).** Dynamic light scattering intensity size distributions are shown for each experimental repeat, TFR (in units of mL min$^{-1}$) of each experiment is shown in the title of each graph. The mean hydrodynamic diameter in Figure 3B is calculated from the hydrodynamic diameter of the most intense peak, which is ~100-200 nm in diameter, and in each case is attributed to vesicle nanocarriers.

*Fluorescent decay rates*

Adapted Förster–Hoffmann equation where fluorescent quantum yield ($\phi_f$) is related to the viscosity (η) by constants (z and α).[1]

$$\phi_f = z\eta^{\alpha}$$

Example fluorescent decay rates for the BODIPY rotor study FRR 1-25.

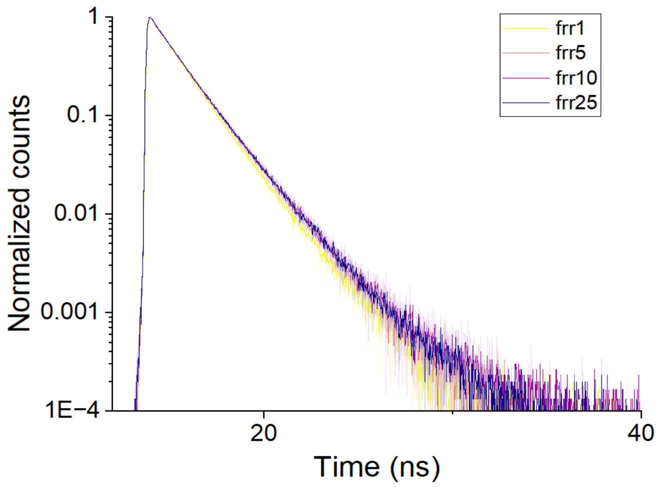


**Figure S6. Fluorescent decay rates for the BODIPY rotor study FRR 1-25.**

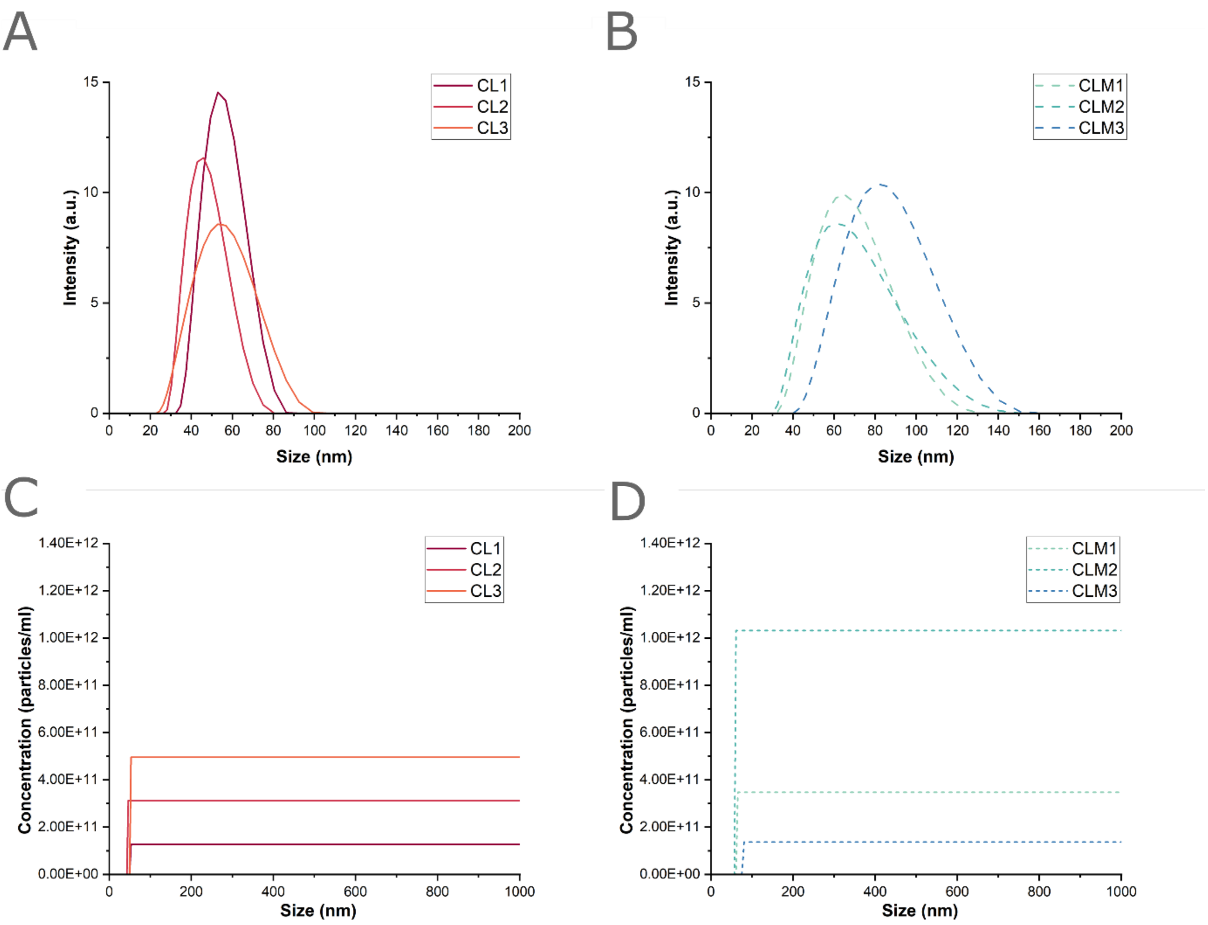


**Figure S7. Characterisation of PEGylated vesicle nanocarriers.** PEGylated vesicle nanocarriers (47:47:6 DOPC:CHOL:DSPEPEG$_{2000}$) can be reliably produced using PMMA VFF devices. Each distribution represents an experimental replicate from separate lipid films dissolved in ethanol, before forming nanocarriers on-chip. **A, C:** Size distribution and particle concentration data for nanocarriers produced without menadione. **B,D:** Size distribution and particle concentration data for nanocarriers produced with menadione.

| | Mean Diameter (nm) | PDI | Particle Concentration (1E11 particles/ml) |
|---|---|---|---|
| **Control Vesicles** | 52.8 ± 2.5 | 0.091 ± 0.02 | 3.12 ± 0.92 |
| **Menadione-loaded control vesicles** | 73.8 ± 5.1 | 0.130 ± 0.01 | 5.05 ± 2.34 |

**Table S3.** Characterisation statistics of PEGylated vesicle nanocarriers. N=3 experimental replicates from separate lipid films dissolved in ethanol, before forming nanocarriers on-chip.

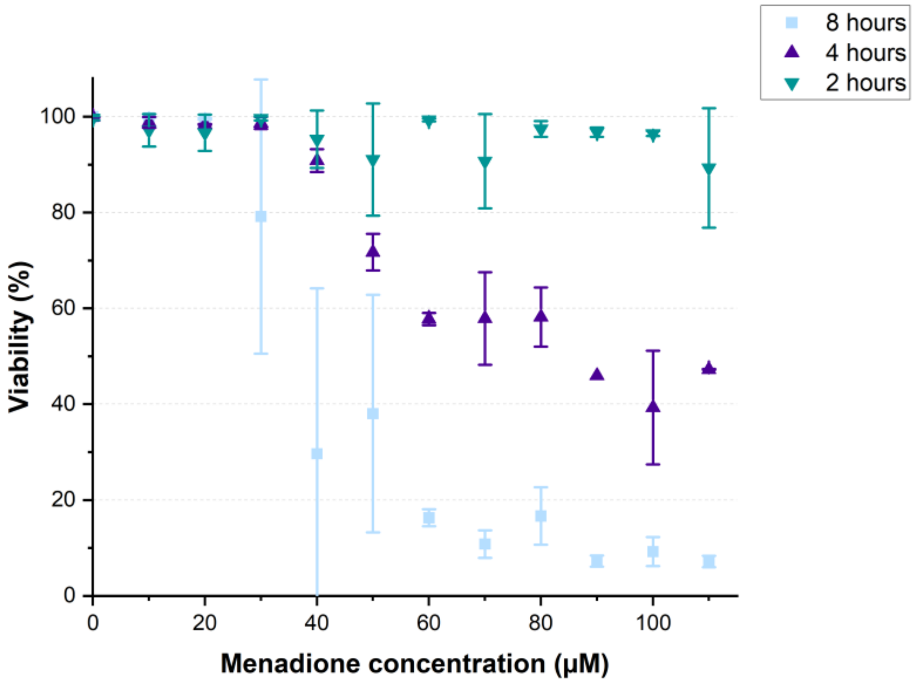


**Figure S8**. **Toxicity of free menadione.** Viability of H9C2 cells treated with 0 – 110 µM menadione solution with a 2 – 8-hour incubation period (n = 6 from 2 biological repeats).

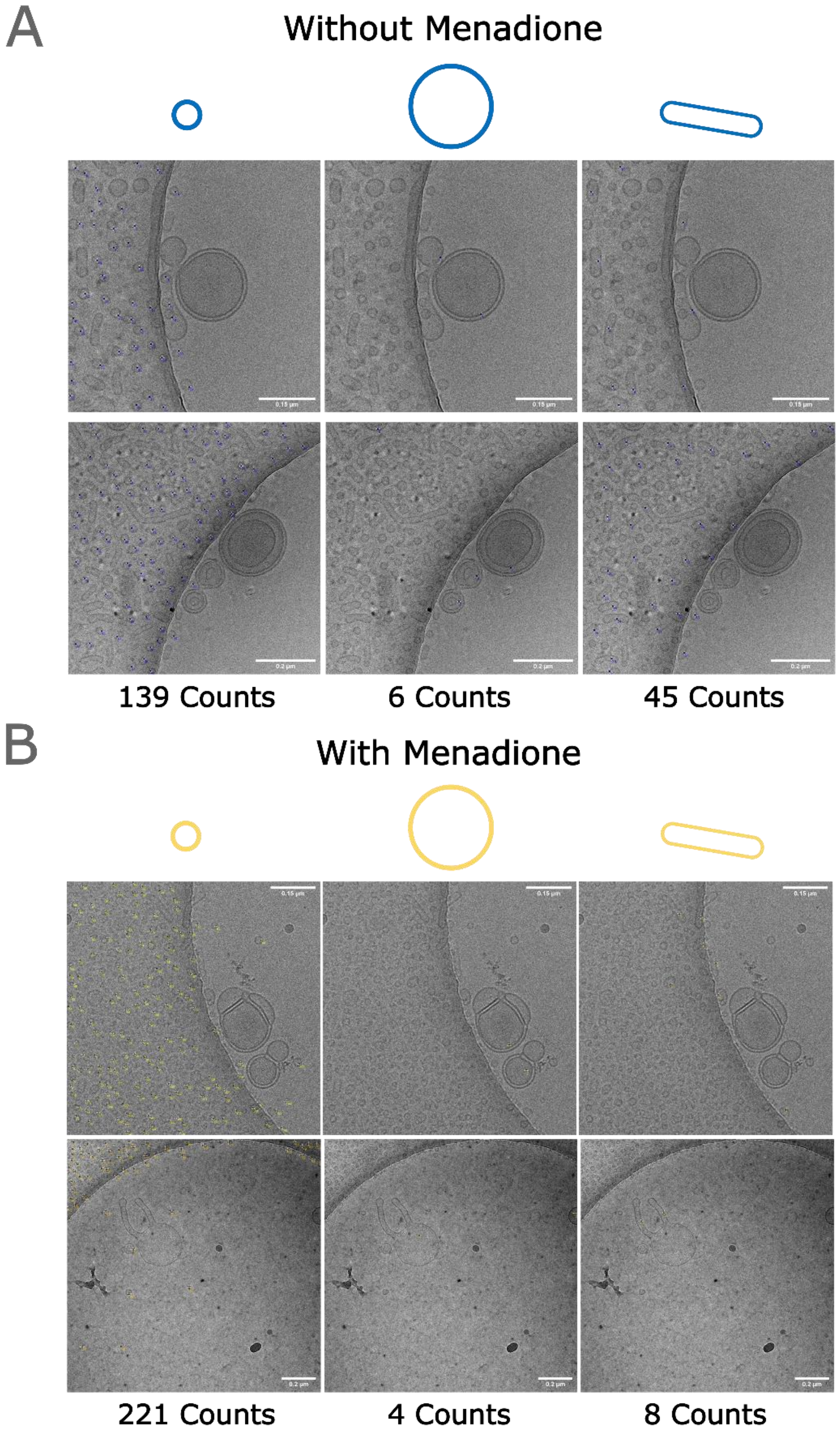
A
Without Menadione
139 Counts
6 Counts
45 Counts
B
With Menadione
221 Counts
4 Counts
8 Counts

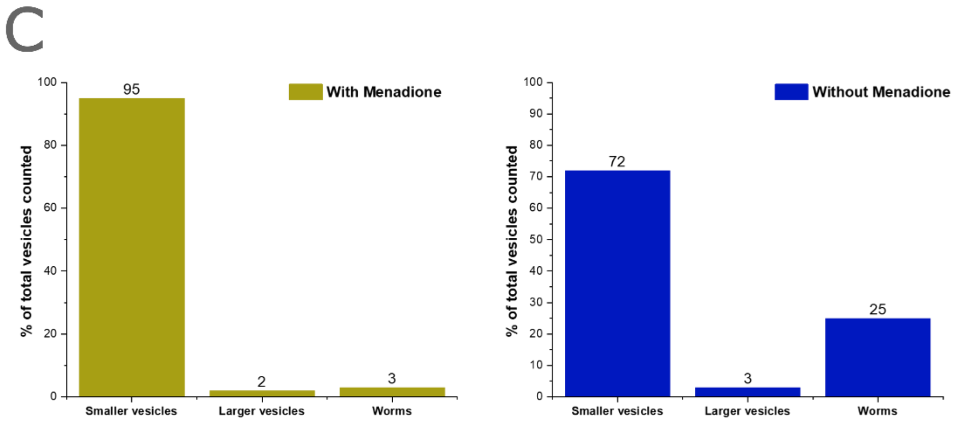


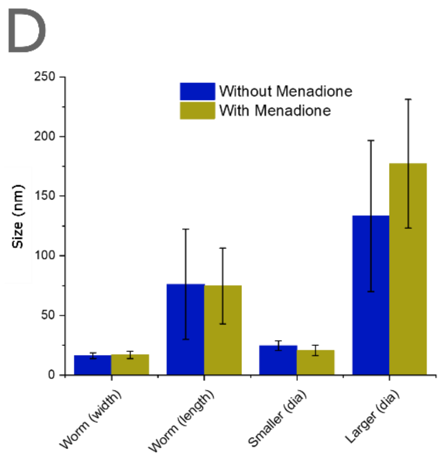


E

| | Without Menadione | | With Menadione | |
|---|---|---|---|---|
| | Size (nm) | Std. dev. (nm) | Size (nm) | Std. dev. (nm) |
| Worm (width) | 16.3125 | 2.41658 | 16.83333 | 3.02306 |
| Worm (length) | 76.125 | 46.26942 | 74.66667 | 31.76301 |
| Smaller Vesicles (dia.) | 24.5 | 4.0466 | 20.66667 | 4.38432 |
| Larger Vesicles (dia.) | 133.4 | 63.30434 | 177.25 | 53.97395 |

**Figure S9. Analysis of cryo-EM images of particles produced by VFF millifluidics. A/B.** Manual classification of particles into smaller spherical shapes (smaller vesicles), larger spherical shapes (larger vesicles) and cylinders (worms) for VFF samples produced without (A) and with (B) menadione (n =190/233 respectively). **C.** Percentage of each particle class with and without menadione respectively. Samples with menadione show very high proportion (95%) of smaller vesicle particles, whilst samples without menadione show sizeable population (25%) of worm-like particles. In each case a small number of larger vesicles can be observed (2-3%). **D.** Sizes of smaller vesicles (diameter), larger vesicles (diameter), and worm-like particles (width and length) for samples with and without menadione. Sizes estimated via manual measurements on ImageJ (n = 81, error bars = 1 standard deviation). **E.** Summary statistics of each particle population with and without menadione (n = 81).

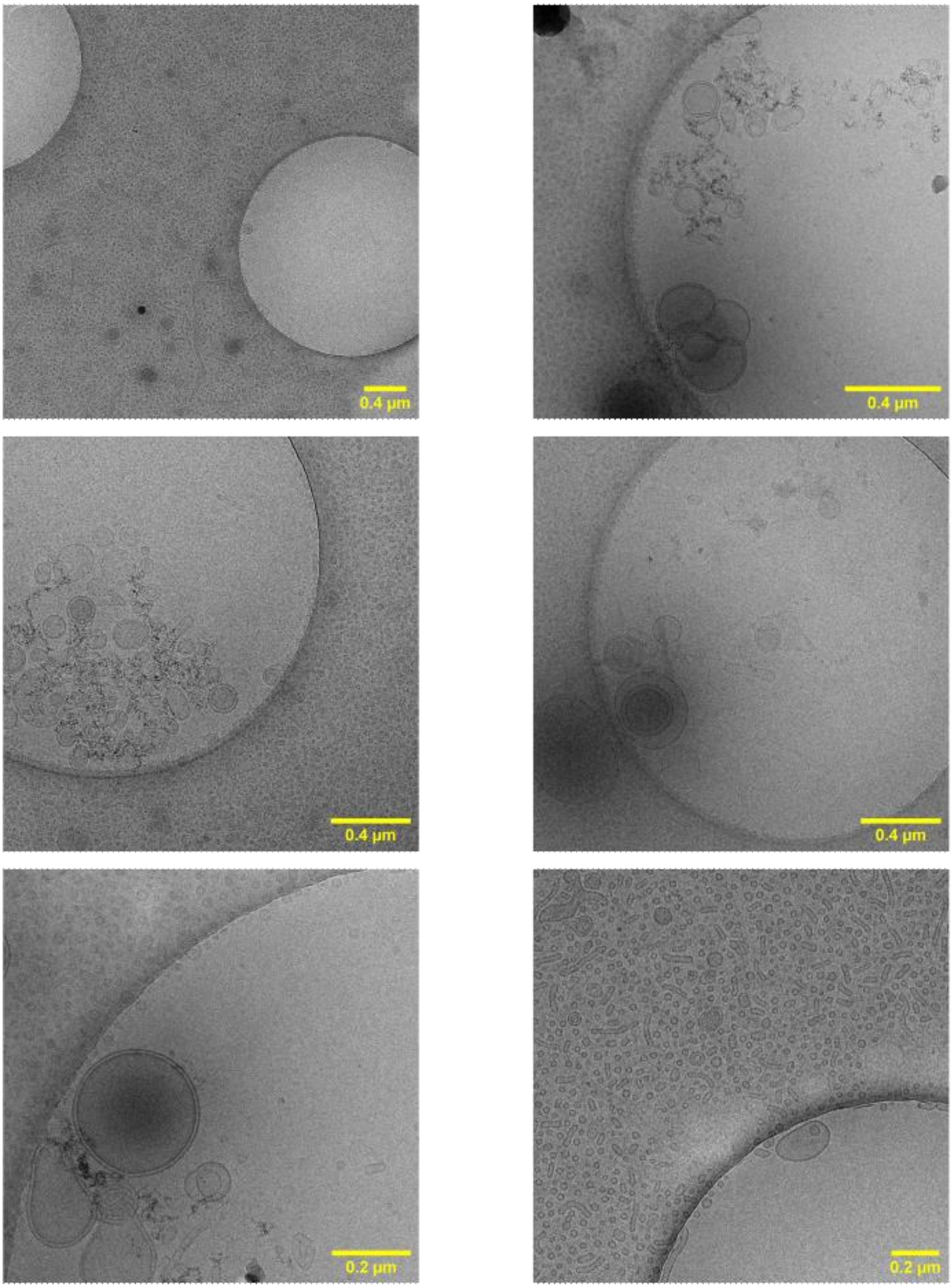
0.4 µm
0.4 µm
0.4 µm
0.4 µm
0.2 µm
0.2 µm

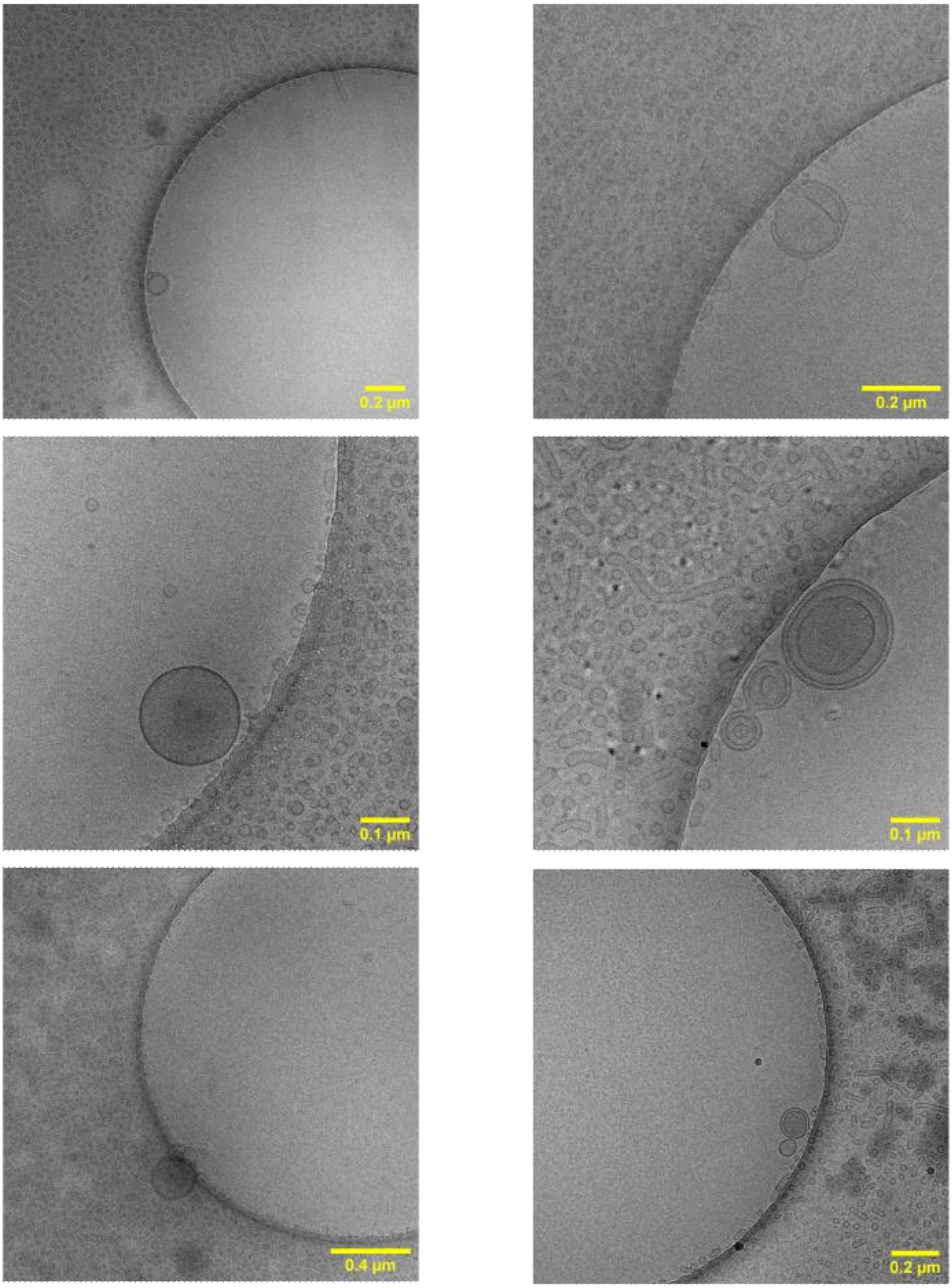
0.2 µm
0.2 µm
0.1 µm
0.1 µm
0.4 µm
0.2 µm

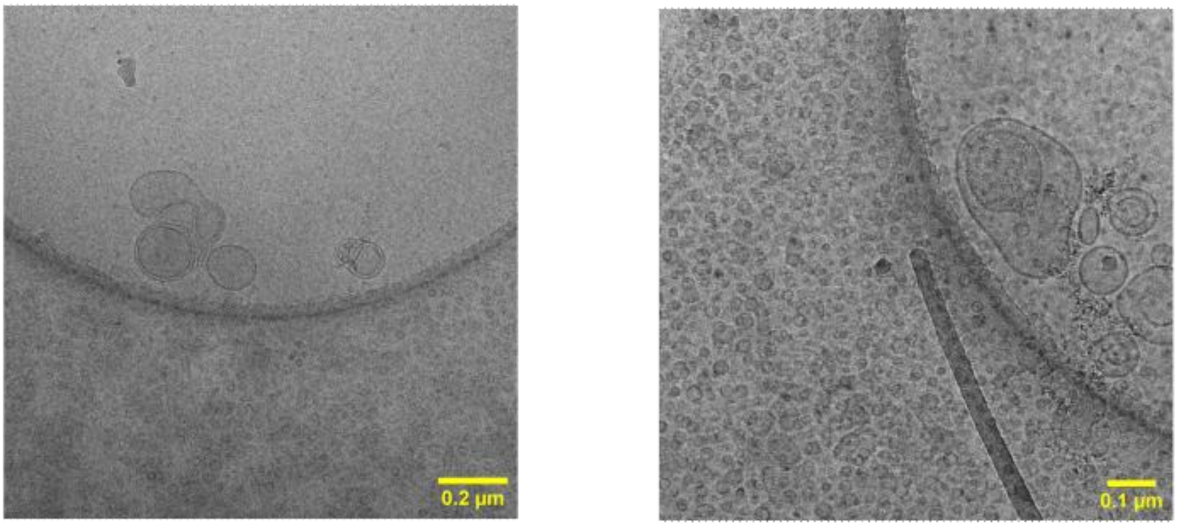


**Figure S10. Compiled cryo-EM images of CL formulation without menadione encapsulation. All** images were taken around the grid section and scale bars are provided for each image.

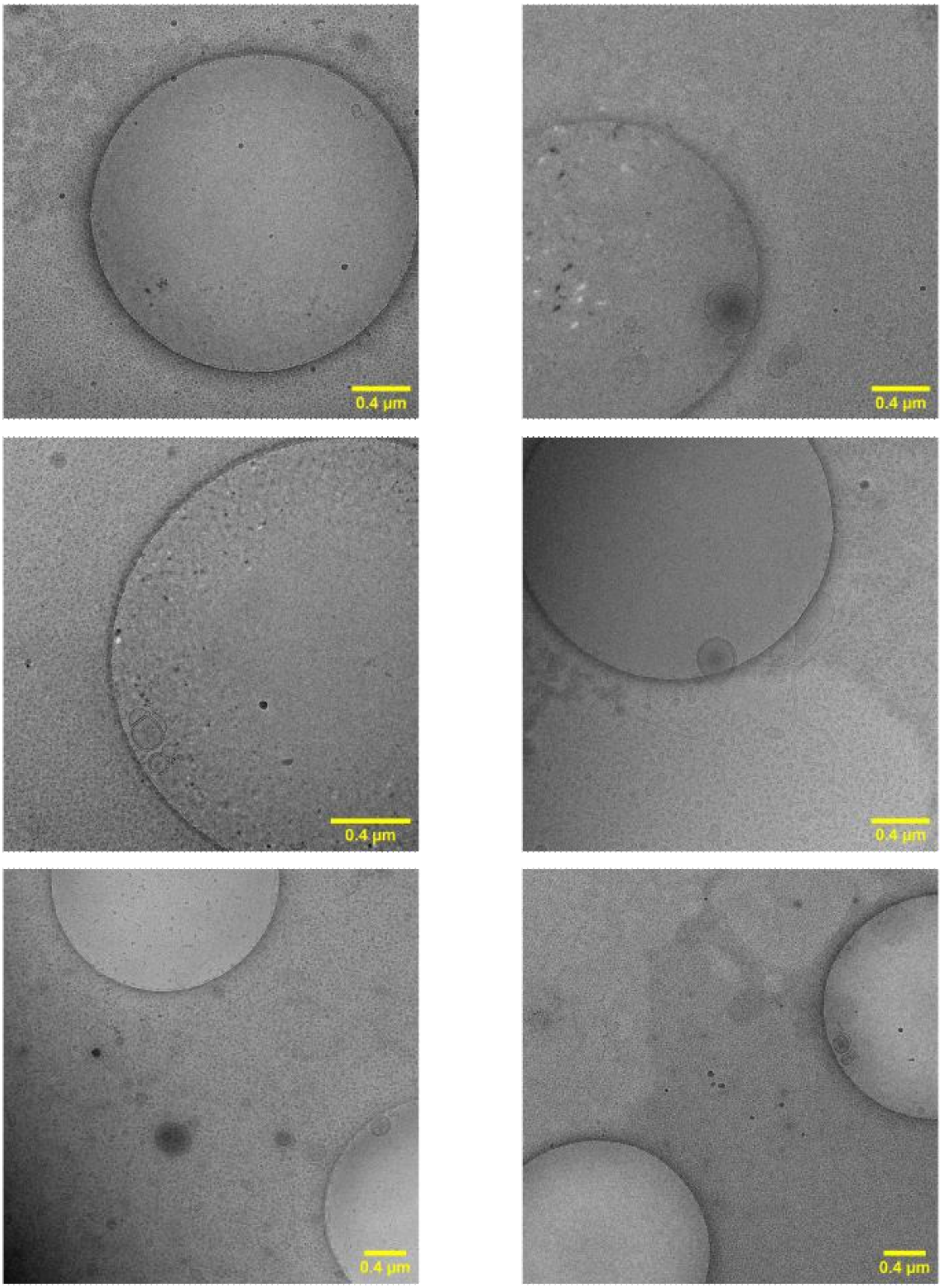
0.4 µm
0.4 µm
0.4 µm
0.4 µm
0.4 µm
0.4 µm

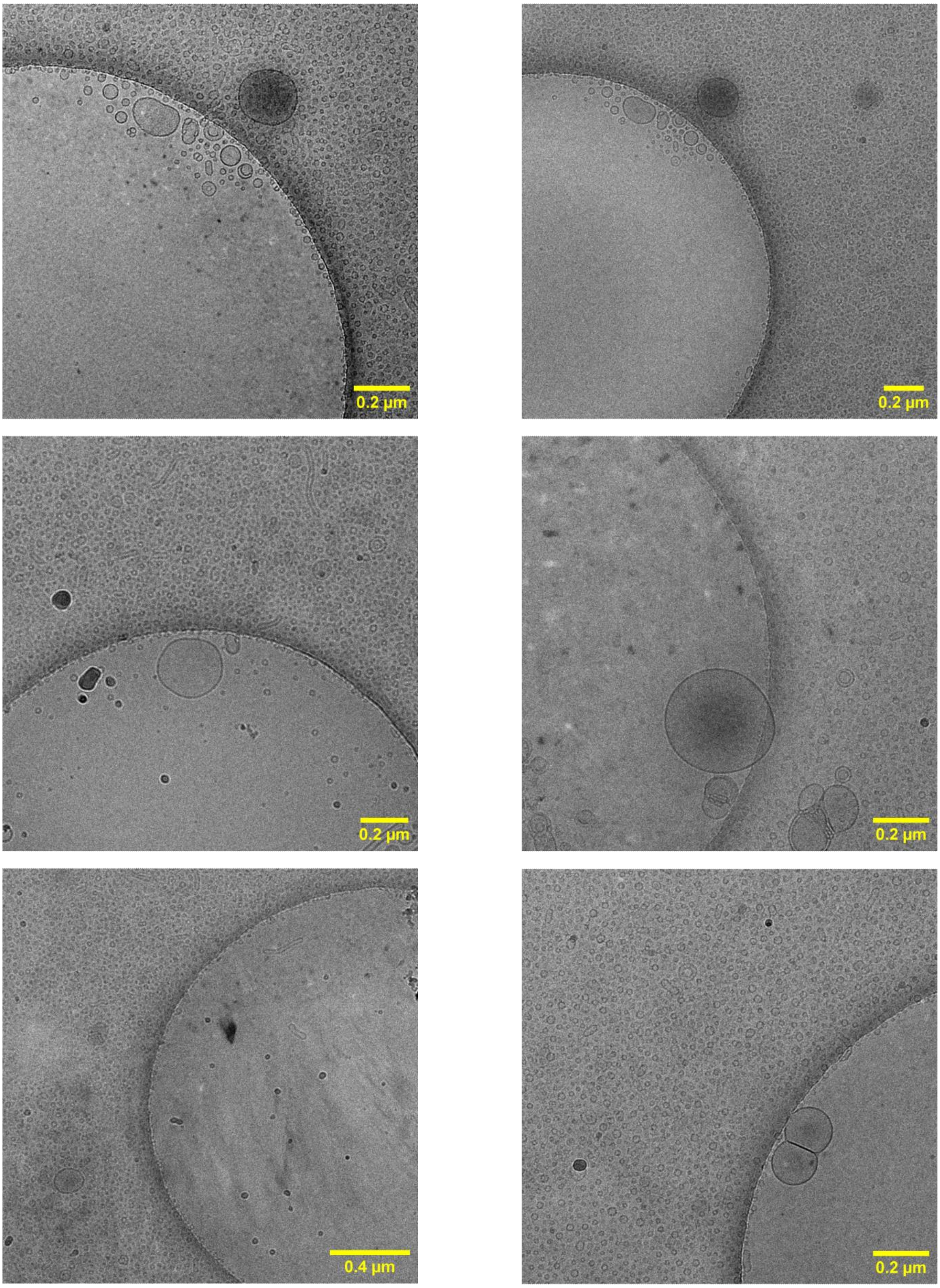
0.2 µm
0.2 µm
0.2 µm
0.2 µm
0.4 µm
0.2 µm

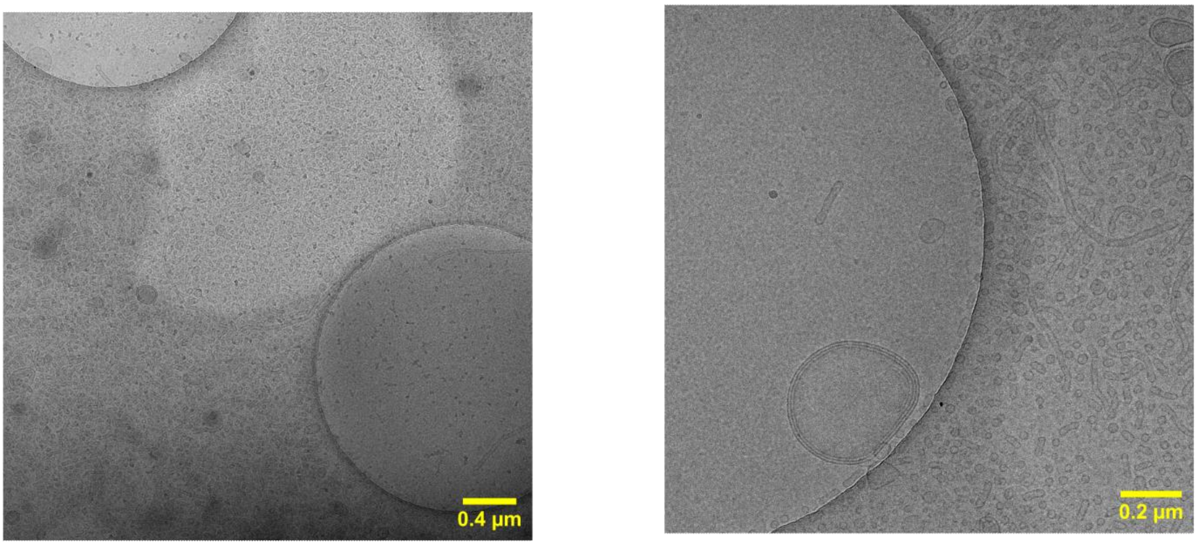


**Figure S11. Compiled cryo-EM images of CL formulation with menadione encapsulation.** All images were taken around the grid section and scale bars are provided for each image.

*X-ray diffraction of CL formulation*

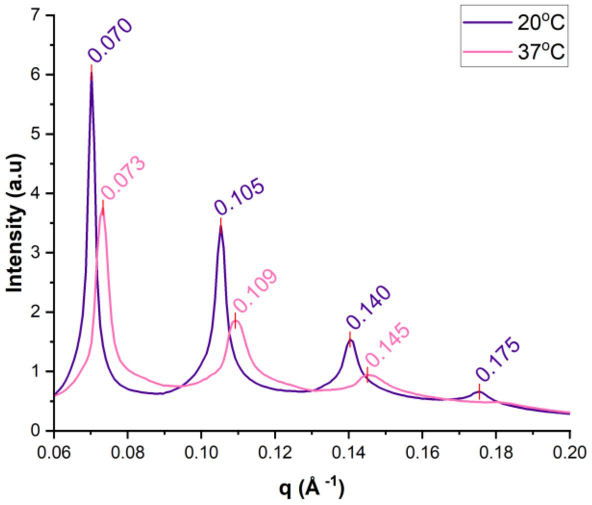


**Figure S12.** Small-angle x-ray scattering was collected at diamond light source I22 for a 10 mg mL$^{-1}$ (47:46:7, DOPC:CHOL:DSPE-PEG$_{2000}$ mol%) sample prepared in PBS using 30 cycles of freeze-thaw homogenization. Peaks index to the 2,3,4,5 L$_{\alpha}$ peaks with a corresponding repeat spacing of 179.5 Å and 172.1 Å, respectively.

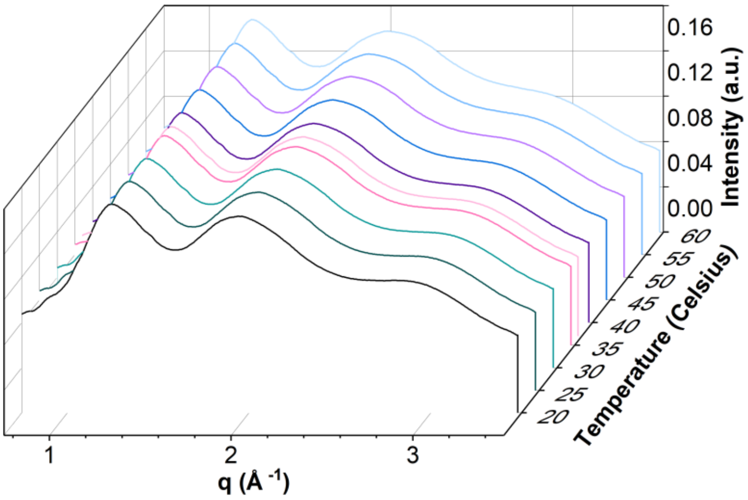


**Figure S13.** Simultaneously the wide-angle x-ray scattering was collected, where polycarbonate and water peaks are visible and the rest is broad, indicating the L$_{\alpha}$ phase.

*Quantifying encapsulation efficiency*

Absorption (Y) values were first used to calculate the menadione concentration (X) using the calibration plot fit.

$$Y = 0.922522 \cdot X \; - 0.04642$$

**Equation S2.** Menadione concentration calculation.

The absorbance maximum was first read from the spectrum of CL liposomes and then converted to menadione concentration ($g_{sample}$ = 0.14 ± 0.01 mg mL$^{-1}$) using the calibration equation (**Figure S9B**). The measured menadione concentration and the sample menadione concentration ($g_{max}$ = 0.40 mg mL$^{-1}$) values were used to calculate encapsulation efficiency (EE) using the following relation.

$$EE\ (\%) = 100\ \cdot \frac{g_{sample}}{g_{max}} = 34.03\ \pm 0.23\ \%$$

**Equation S3.** Encapsulation efficiency calculation.

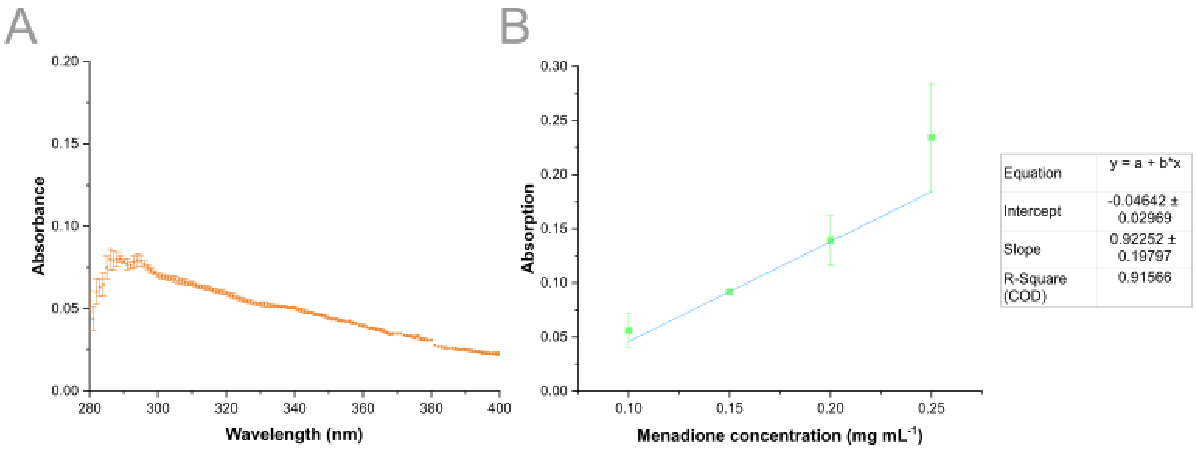


**Figure S14. A)** Absorption spectrum of CL liposomes (1 mg mL$^{-1}$ lipid) containing menadione (0.4 mg mL$^{-1}$) post-VFF (n = 3). **B)** Absorption spectra of menadione standards in PBS at 340 nm[2], used for the calibration plot (n=3).

*H9C2 viability assay*

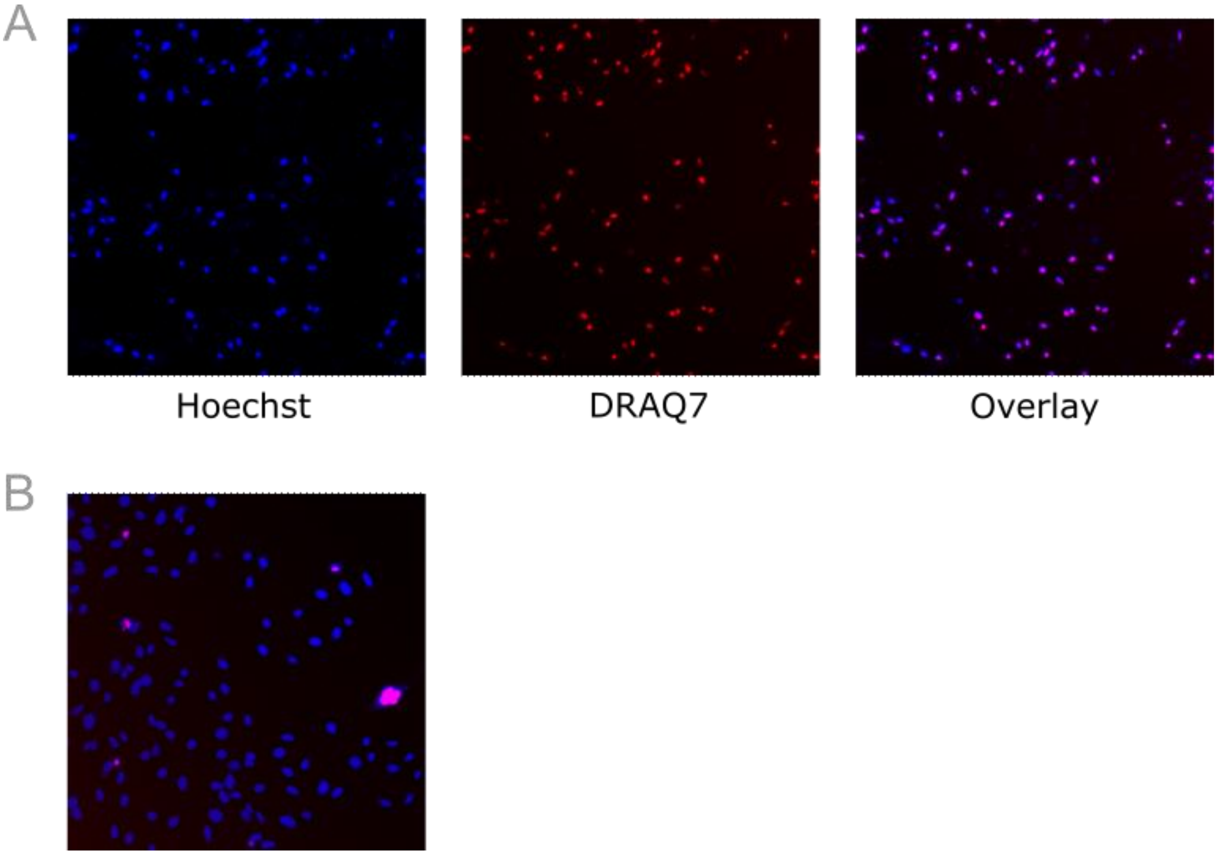


**Figure S15. Example images of cell stains used for the viability assay. A)** Hoechst image of high cell death samples (left), DRAQ7 image of high cell death samples (center), and the overlay of both channels (right). **B)** Overlay of Hoechst and DRAQ7 channels for a low cell death sample.

| Parameter | PMMA VFF Device (This study) | COC VFF Device (Hood *et al*, 2015)[3] | DLP VFF Device (Chen *et al*, 2019)[4] |
|---|---|---|---|
| **Maximum Total Flow Rate Tested (ml/min)** | 2.1 | 4.5 | 30 |
| **Production Rate (mg/hour lipid)** | 105 (5 mg/ml, 6.35 mM) | 90 (20 mM) | 235 (5 mM) |
| **Aspect Ratio** | 50 | 100 | 40 |

**Table S4.** Comparison of the VFF device produced in this study versus existing technologies.

## References


[1] Y. Wu, M. Štefl, A. Olzyńska, M. Hof, G. Yahioglu, P. Yip, D. R. Casey, O. Ces, J. Humpolíčková, M. K. Kuimova, *Physical Chemistry Chemical Physics* **2013**, *15*, 14986.

[2] B. R. Acharya, D. Choudhury, A. Das, G. Chakrabarti, *Biochemistry* **2009**, *48*, 6963.

[3] R. R. Hood, D. L. Devoe, *Small* **2015**, *11*, 5790.

[4] Z. Chen, J. Y. Han, L. Shumate, R. Fedak, D. L. DeVoe, *Adv Mater Technol* **2019**, *4*, 1.
